# Legionella longbeachae Regulates the Association of Polyubiquitinated Proteins on Bacterial Phagosome with Multiple Deubiquitinases

**DOI:** 10.1128/spectrum.04179-22

**Published:** 2023-02-15

**Authors:** Yunjia Shi, Hongtao Liu, Kelong Ma, Zhao-Qing Luo, Jiazhang Qiu

**Affiliations:** a State Key Laboratory for Zoonotic Diseases, Key Laboratory for Zoonosis Research of the Ministry of Education, College of Veterinary Medicine, Jilin University, Center for Pathogen Biology and Infectious Diseases, The First Hospital of Jilin University, Changchun, China; b Purdue Institute for Inflammation, Immunology and Infectious Disease and Department of Biological Sciences, Purdue University, West Lafayette, Indiana, USA; South China Sea Institute of Oceanology

**Keywords:** *Legionella*, type IV secretion system, effector protein, deubiquitinase, ubiquitination

## Abstract

*Legionella* spp. are the causative agents of a severe pneumonia known as Legionnaires’ disease. Upon being engulfed by host cells, these environmental bacteria replicate intracellularly in a plasma membrane-derived niche termed *Legionella*-containing vacuole (LCV) in a way that requires the defective in organelle trafficking/intracellular multiplication (Dot/Icm) protein transporter. Our understanding of interactions between *Legionella* and its hosts was mostly based on studies of Legionella pneumophila. In this study, we found that the LCVs created by virulent Legionella longbeachae are similarly decorated by polyubiquitinated proteins to those formed by L. pneumophila and that the ubiquitin-proteasome system (UPS) is required for optimal intracellular growth of L. longbeachae. Furthermore, we utilized bioinformatics methods and the ubiquitin-vinylmethyl ester probe to obtain potential deubiquitinases (DUBs) encoded by L. longbeachae. These efforts led to the identification of 9 L. longbeachae DUBs that displayed distinct specificity toward ubiquitin chain types. Among these, LLO_1014 and LLO_2238 are associated with the LCVs and impact the accumulation of polyubiquitinated species on the bacterial phagosome. Moreover, LLO_1014 and LLO_2238 could fully restore the phenotypes associated with Δ*ceg23* (*lotB*) and Δ*lem27* (*lotC*) mutants of L. pneumophila, indicating that these DUBs have similar functions. Together, these results reveal that L. longbeachae uses multiple DUBs to construct an intracellular niche for its replication.

**IMPORTANCE** *Legionella* spp. are opportunistic intracellular bacterial pathogens that cause Legionnaires’ disease. *Legionella* utilizes the Dot/Icm type IV secretion system to deliver effector protein into host cells to modulate various cellular functions. At least 26 L. pneumophila effectors are known to hijack the host ubiquitin system via diverse mechanisms. L. longbeachae is the second leading cause of Legionnaires’ disease worldwide. However, our knowledge about the interactions between L. longbeachae and its hosts is very limited. Here, we found that, similar to L. pneumophila infection, the host ubiquitin proteasome system is also important for the intracellular replication of L. longbeachae. In addition, the bacterial phagosomes harboring L. longbeachae are enriched with polyubiquitinated proteins in a Dot/Icm system-dependent manner. We further identified 9 L. longbeachae proteins that function as DUBs with distinct ubiquitin chain specificity. Of note, several of the phagosome-associated L. longbeachae DUBs regulate the recruitment of polyubiquitinated proteins to the LCV.

## INTRODUCTION

Ubiquitination is one of the most important posttranslational modifications (PTMs) in eukaryotes and is involved in the regulation of almost all cellular events (1, 2). The ubiquitin (Ub) molecule is covalently attached to the lysine residues of target proteins via isopeptide bonds through a reaction cascade involved in three enzymes, an E1 Ub-activating enzyme, an E2 Ub-conjugating enzyme, and an E3 Ub ligase (1). Substrates can be conjugated with a single Ub molecule on a single residue (mono-ubiquitination) or multiple sites (multimono-ubiquitination) (3). In addition, Ub itself can be further ubiquitinated at one of its lysine residues (K6, K11, K27, K29, K33, K48, K63) or N-terminal methionine (M1) to generate at least eight poly-Ub chains (3). The different ubiquitination types result in distinct functional outcomes of substrates. For example, proteins modified by K48-linked polyubiquitin chains often are targeted for proteasomal degradation, whereas K63-linked polyubiquitination mostly plays critical roles in cellular signaling such as immunity and DNA damage responses (2). To date, over 600 E3 ligases have been identified in human cells (4).

Akin to most well-studied PTMs, protein ubiquitination is a reversible process. Removal of Ub from modified proteins is carried out by a family of protease termed deubiquitinase (DUB), which specifically catalyzes the hydrolysis of isopeptide linkages in poly-Ub chains or those linking Ub to target substrates, leading to the liberation of the substrate protein and recycling of Ub (5). To date, approximately 100 DUBs have been reported in humans, which are classified into 7 families according to their distinct structures and modes of action (5). These include Ub-specific proteases (USPs), Ub carboxyl-terminal hydrolases (UCHs), ovarian tumor domain proteases (OTUs), Machado-Joseph domain proteases (MJDs), motif interacting with ubiquitin-containing novel DUB family (MINDY), zinc finger with UFM1-specific peptidase domain protein (ZUFSP), and JAB1/MPN/MOV34 proteases (JAMMs) (5). Except for JAMMs, which are zinc-dependent metalloproteases, other DUB families are cysteine proteases (5, 6).

Although the ubiquitination machinery is unique to eukaryotic organisms, it is frequently exploited by pathogens via virulence proteins (effectors) to counteract host immune defense and promote their proliferation (7). These effectors often behave as mimics of E3 ligases and DUBs to interfere with host Ub signaling pathways via targeting of specific host substrates. For instance, the Shigella flexneri effector IpaH9.8 is a bacterial E3 ligase that targets human guanylate binding protein 1 (hGBP1) for degradation via K48-linked ubiquitination, thereby disarming hGBP1-mediated host antimicrobial defense and promoting virulence (8). Salmonella enterica serovar Typhimurium (*S.* Typhimurium) effector SopA, as a HECT E3 ubiquitin ligase, can ubiquitinate two host E3 ligases, TRIM65 and TRIM56, to block interferon alpha (IFN-α)-mediated immune response (9). Similarly, virulence factors harboring DUB activity have been found in pathogenic bacteria, including *S.* Typhimurium, Chlamydia trachomatis, and Chlamydia pneumonia (10). SseL secreted by *S.* Typhimurium belongs to the CE clan of proteases capable of hydrolyzing poly-Ub chains with a preference for K63 linkage (11). SseL inhibits host selective autophagy through deubiquitinating the *S.* Typhimurium-induced ubiquitinated aggregates and aggresome-like induced structures (ALIS) (12, 13). Cdu1 and Cdu2 from C. trachomatis are DUBs that function to counteract NF-κB activation by deubiquitinating IκBα to facilitate bacterial survival in host cells (14).

*Legionella* spp. are opportunistic intracellular bacterial pathogens ubiquitously found in aquatic and soil environments (15). Upon inhalation of contaminated aerosols, these bacteria colonize and replicate in human alveolar macrophages and cause a severe atypical pneumonia known as Legionnaires’ disease (16). To date, over 65 *Legionella* species have been identified, with about 30 of them reported to associate with human disease (17). Although most cases of Legionnaires’ disease are attributed to Legionella pneumophila, diagnosed cases that resulted from other *Legionella* species are increasing (17). Among these, Legionella longbeachae accounts for 50 to 60% of cases in New Zealand and Australia, and cases caused by this species in Europe and several countries in Asia have also been increasingly reported during the past decade (18, 19). Although the diseases inflicted by L. longbeachae and L. pneumophila are indistinguishable, these two species exhibit distinct features in environmental niche, physiology, and genetic content (20). L. pneumophila is mainly present in natural and synthetic aquatic environments, whereas L. longbeachae is predominantly found in compost and potting soils (15). Compared to L. pneumophila, L. longbeachae encodes a capsule but lacks flagella, which partly explains the differences in susceptibility of mouse to these species (20, 21). In addition, L. pneumophila displays a pronounced biphasic life cycle that consists of a noninfective replicative phase and an infectious transmissive phase (22). Each of the biphasic phases is characterized by the expression of specific traits that can be reflected by the alteration in gene expression program of *in vitro*-cultured L. pneumophila at the exponential- and postexponential-growth phases (20). Transcriptomic analysis revealed a less prominent alteration of lifestyle in L. longbeachae than in L. pneumophila, suggesting a growth phase-independent virulence strategy employed by L. longbeachae (20).

*Legionella* spp. survive and replicate intracellularly in various host cells, including amoebae and mammalian cells within a membrane-enclosed compartment known as *Legionella*-containing vacuole (LCV) (16). Biogenesis of the LCV requires the defective in organelle trafficking/intracellular multiplication (Dot/Icm) type IV secretion system, which is highly conserved among different species (23, 24). This apparatus injects a large cohort of effectors into the cytosol of infected cells, thereby altering a variety of host signaling pathways to build the LCV that supports intracellular bacterial replication (25). For L. pneumophila, approximately 330 effector proteins have been identified to date, and many of them have been characterized that function to interfere with host processes such as vesicle trafficking, autophagy, ubiquitin signaling, and phosphoinositide metabolism (23, 25).

The ubiquitin network is critical for L. pneumophila virulence, as demonstrated by the fact that vacuoles containing virulent L. pneumophila are highly decorated with polyubiquitinated proteins, and interference of the ubiquitin proteasome system impairs intracellular bacterial growth (26, 27). In line with this, a cohort of L. pneumophila effectors have been characterized as E3 ligases or DUBs, either through the adoption of classical catalytic domains or employment of novel mechanisms (28). Recent comparative genomic analyses of *Legionella* have revealed significant genetic variations, particularly in the effector repertoires among different species (24, 29). L. longbeachae is predicted to code for 110 Dot/Icm substrates (20). Strikingly, over 66% of the reported L. pneumophila effector proteins are absent from L. longbeachae, and 51 unique substrates have been identified (20). Despite these differences, a recent study demonstrates that both species develop phenotypically similar replicative vacuoles in host cells (30). Hence, different *Legionella* species might utilize distinct cohorts of effectors to hijack cellular functions in similar ways. In the present study, we first observed that the LCV formed by L. longbeachae is similarly decorated with polyubiquitinated proteins as L. pneumophila. Then, we systematically identify DUBs encoded by L. longbeachae and demonstrate that several of these enzymes play roles in the modulation of polyubiquitinated proteins on the phagosomal membrane.

## RESULTS

### The ubiquitin proteasome system is important for intracellular replication of L. longbeachae.

**FIG 1:**
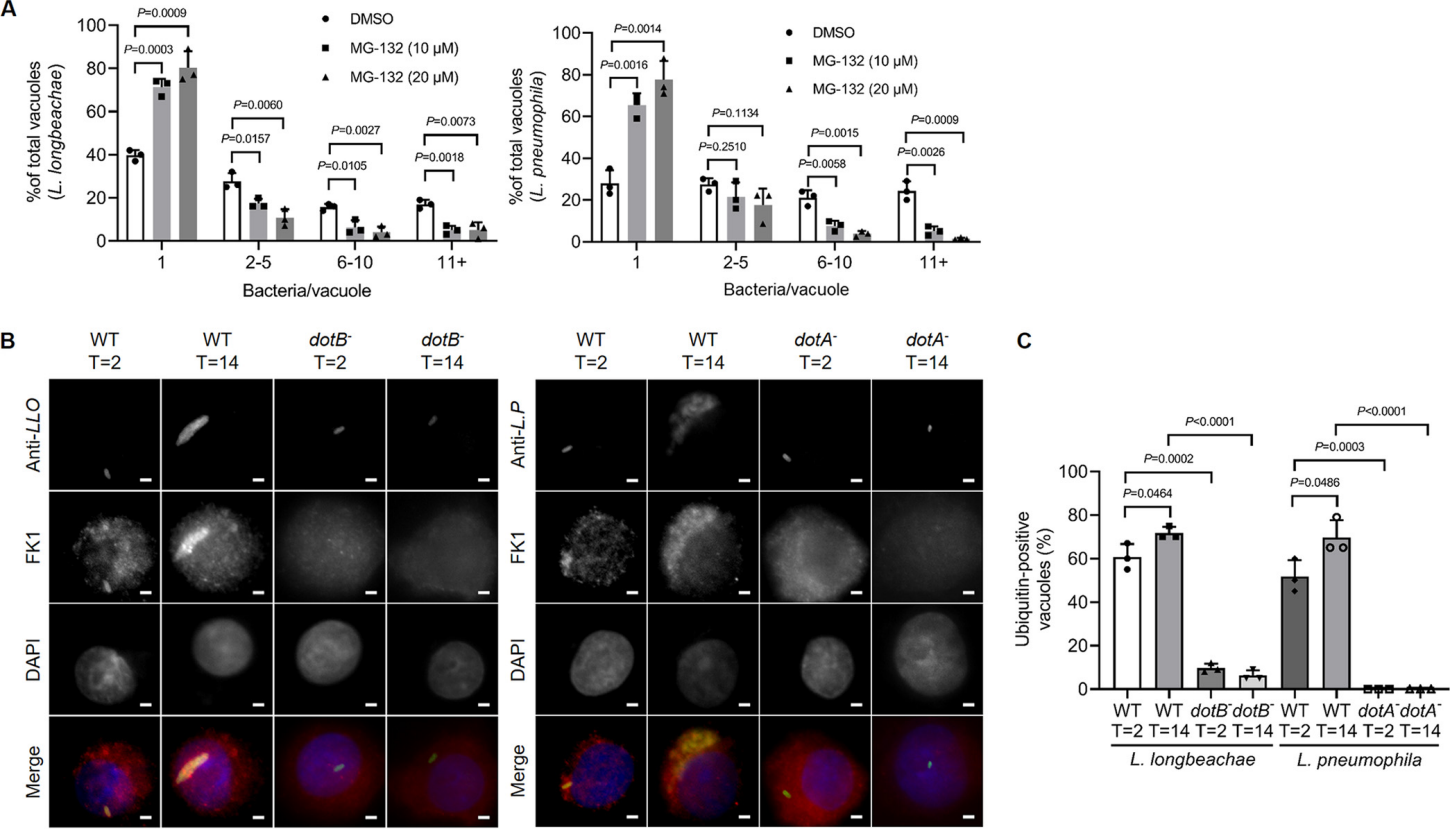
The proteasome is required for optimal intracellular growth of L. longbeachae and Dot/Icm-dependent decoration of its phagosomes with polyubiqutinated proteins. (A) U937 cells pretreated with the indicated concentrations of MG-132 for 1 h were challenged with wild-type L. longbeachae or L. pneumophila at an MOI of 10 for 2 h. After removing extracellular bacteria, infections proceeded for 14 h. The number of bacteria in each vacuole was determined using a fluorescence microscope after immunostaining with the L. longbeachae or L. pneumophila antibody. At least 100 vacuoles were scored for each sample. (B) U937 cells were infected wild-type or the Δ*dotB* mutant L. longbeachae and L. pneumophila strains for the indicated durations. Fixed cells were then immunostained with anti-L. longbeachae, anti-L. pneumophila, and anti-ubiquitinated proteins (FK1). The nuclei were stained with DAPI (4′,6-diamidino-2-phenylindole) (dark blue). The association of polyubiquitinated species with the phagosomes was scored under a fluorescence microscope. Scale bar, 2 μm. (C) Percentages of phagosomes associated with polyubiquitinated proteins. At least 100 LCVs per coverslip were measured for each sample. Results in panels A and C are means ± standard derivations (SDs) calculated from three coverslips. Similar results were obtained in three independent experiments. Statistics analysis was performed by unpaired two-tailed Student *t* tests, and a *P* value of <0.05 represents a significant difference.

It has been established that a functional ubiquitin-proteasome system (UPS) is required for optimal intracellular replication of L. pneumophila (26, 27). Moreover, polyubiquitinated proteins are enriched on the LCV harboring virulent L. pneumophila throughout its intracellular life cycle (26). To determine whether the UPS system is also important for L. longbeachae virulence, we treated U937 cells with the proteasome inhibitor MG-132 for 1 h prior to bacterial infection. Then, the cells were infected with wild-type (WT) L. longbeachae, and the bacterial number in each vacuole was examined after 14 h of infection. We found that the percentage of nonreplicative LCVs was significantly increased in cells receiving each of the testing concentrations of MG-132, which was accompanied by a marked reduction of medium and large replicative vacuoles that harbor 2 to 10 and ≥11 bacteria, respectively (Fig. 1A). As a control, similar observations were found in cells infected with L. pneumophila (Fig. 1A). Thus, inhibition of the UPS interferes with intracellular growth of L. longbeachae.

Next, we challenged U937 cells with L. longbeachae and immunostained with an antibody against ubiquitinated proteins. At 2 h postinfection, polyubiquitinated species were associated with 60% of LCVs bearing WT L. longbeachae (Fig. 1B and C). In contrast, only 9% of ubiquitin-labeled vacuoles were inspected in cells infected with the *dotB^−^* mutant (Fig. 1B and C), suggesting the requirement of effectors for the recruitment of ubiquitinated proteins to the LCV. Decoration of the LCV by ubiquitin appears to be persistent throughout the infection cycle, as 71% of the LCVs stained positively for polyubiquitinated proteins 14 h after bacterial uptake (Fig. 1B and C). As a control, a similar association of the LCVs with polyubiquitinated species was observed in cells challenged with L. pneumophila strains (Fig. 1B and C). Taken together, our results suggest that L. longbeachae utilizes mechanisms similar to those of L. pneumophila to hijack the host ubiquitin system for the construction of its LCV.

### Identification of DUBs from L. longbeachae.

**FIG 2:**
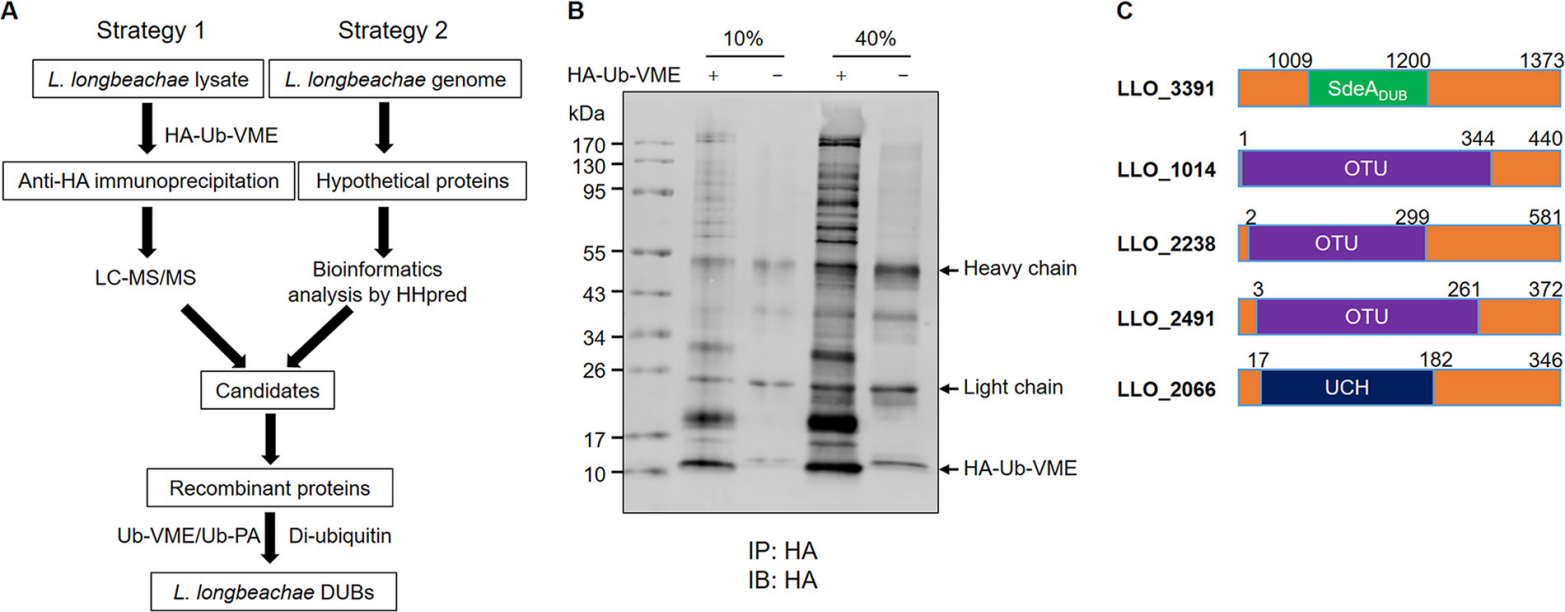
Identification of DUBs in L. longbeachae. (A) Diagram of the strategies used to identify DUBs from L. longbeachae. (B) Lysates of L. longbeachae were incubated with HA-Ub-VME for 2 h at 37°C. Following immunoprecipitation (IP) with anti-HA agarose, HA-Ub-VME-reactive proteins were probed by Western blotting with an HA-specific antibody. Ten percent and 40% indicate the amount of anti-HA IP products loaded on the gel. (C) Predicted DUB domain of the candidate DUBs identified by bioinformatics analysis using HHpred. Results in panel B are one representative from three independent experiments with similar results.

At least 26 L. pneumophila Dot/Icm substrates have been demonstrated to interfere with the host ubiquitin system via diverse mechanisms, such as molecular mimicry of E3 ubiquitin ligases or DUBs (31). We employed two independent strategies to identify putative DUBs from L. longbeachae (Fig. 2A). First, we used the hemagglutinin (HA)-tagged Ub-vinylmethyl ester (HA-Ub-VME), an activity-based DUB probe that is able to covalently attach to the nucleophilic catalytic cysteine residue of DUBs (32). Incubation of HA-Ub-VME with cell lysates of L. longbeachae generated multiple Ub-VME-modified protein bands (Fig. 2B), indicating effective capture of putative DUBs by this probe. The proteins retained by anti-HA agarose beads were subsequently identified by mass spectrometric analysis (see Data Set S1 in the supplemental material). Members of the SidE family in L. longbeachae showed the highest scores among the candidates, which is consistent with results from our earlier experiments using lysates of L. pneumophila (Data Set S1; Table 1) (33). Despite having lower protein scores, several uncharacterized proteins were also identified (Table 1). The second method involved the use of comprehensive bioinformatics analysis of all hypothetical proteins in L. longbeachae via pairwise comparison of profile-hidden Markov models (HHpred) (34). These efforts allowed us to identify a total of 11 proteins possessing homology to known DUBs, including five SidE homologue proteins. Additionally, a fragment of LLO_3391 ranging from residues 1009 to 1200 shows homology to the SidE DUB domain; LLO_1104, LLO_2238, and LLO_2491 are distantly similar to members of the OTU family DUBs, and LLO_2066 belongs to DUBs of the UCH family (Fig. 2C and Table 2). Except for LLO_3391, all other candidates have homologous counterparts in L. pneumophila with considerable sequence similarities.

**TABLE 1:** Mass spectrometric analysis of canonical deubiquitination activity effectors in L. longbeachae

Protein name	Gene name	MW^*a*^ (kDa)	Protein score	Sequence coverage (%)	No. of unique peptides	No. of peptides	Ub-VME no. of PSMs	Control no. of PSMs
Uncharacterized protein	LLO_1369	87.53	104.19	4.40	3	3	3	
Putative coiled coil protein	LLO_1631	81.93	499.41	21.52	15	15	15	8
Uncharacterized protein	LLO_0794	127.73	719.54	16.88	17	17	18	10
Uncharacterized protein	LLO_3118	31.60	513.91	52.46	10	10	11	5
Uncharacterized protein	LLO_2210	89.89	127.53	5.70	5	5	5	
Uncharacterized protein	LLO_2179	59.59	701.41	30.60	17	17	20	5
Uncharacterized protein	LLO_2985	60.15	997.52	34.80	17	19	25	10
Putative coiled-coil protein, similar to eukaryotic protein	LLO_2313	209.85	98.44	1.93	2	3	3	
Uncharacterized protein	LLO_2238	67.45	189.76	10.33	6	6	6	
Homologous to SidE substrate of Dot/Icm secretion system	LLO_0424	169.36	1,710.78	31.56	25	41	49	8
Homologous to SidE substrate of Dot/Icm secretion system	LLO_0426	176.37	5,386.95	65.04	75	94	134	13
Homologous to SidE substrate of Dot/Icm secretion system	LLO_3092	177.83	3,845.64	55.30	61	76	99	5
Similar to Sid proteins	LLO_3095	169.01	4,171.46	61.16	7	82	103	

a MW, molecular weight.

**TABLE 2:** Bioinformatics analysis of hypothetical proteins via HHpred

L. longbeachae proteins		Target proteins				
Gene no.	Aligned region	Name	Aligned region	Probability (%)	Identity (%)	PDB accession no.
LLO_3391	1009−1200	SdeA deubiquitinase, *Legionella*	4−192	100	58	5CRB_A
LLO_2238	2–299	Deubiquitinase, *Legionella*, OTU, effector protein Lpg2529	2−297	100	65	6YK8_A
LLO_1014	1–344	Type IV secretion protein Dot Ceg23, ubiquitin thioesterase OTU1	1−351	100	63	6KS5_B
LLO_2491	3–261	Deubiquitinase, *Legionella*, OTU, effector protein Lpg2529	1−297	100	20	6YK8_A
LLO_0075a	3−144	Ubiquitin thioesterase OTUB1	44−203	98.79	11	2ZFY_A
LLO_2066	17−182	Deubiquitinase SseL, UCH family	4−161	97.22	14	5UBW_B

### Validation of the potential L. longbeachae DUBs.

**FIG 3:**
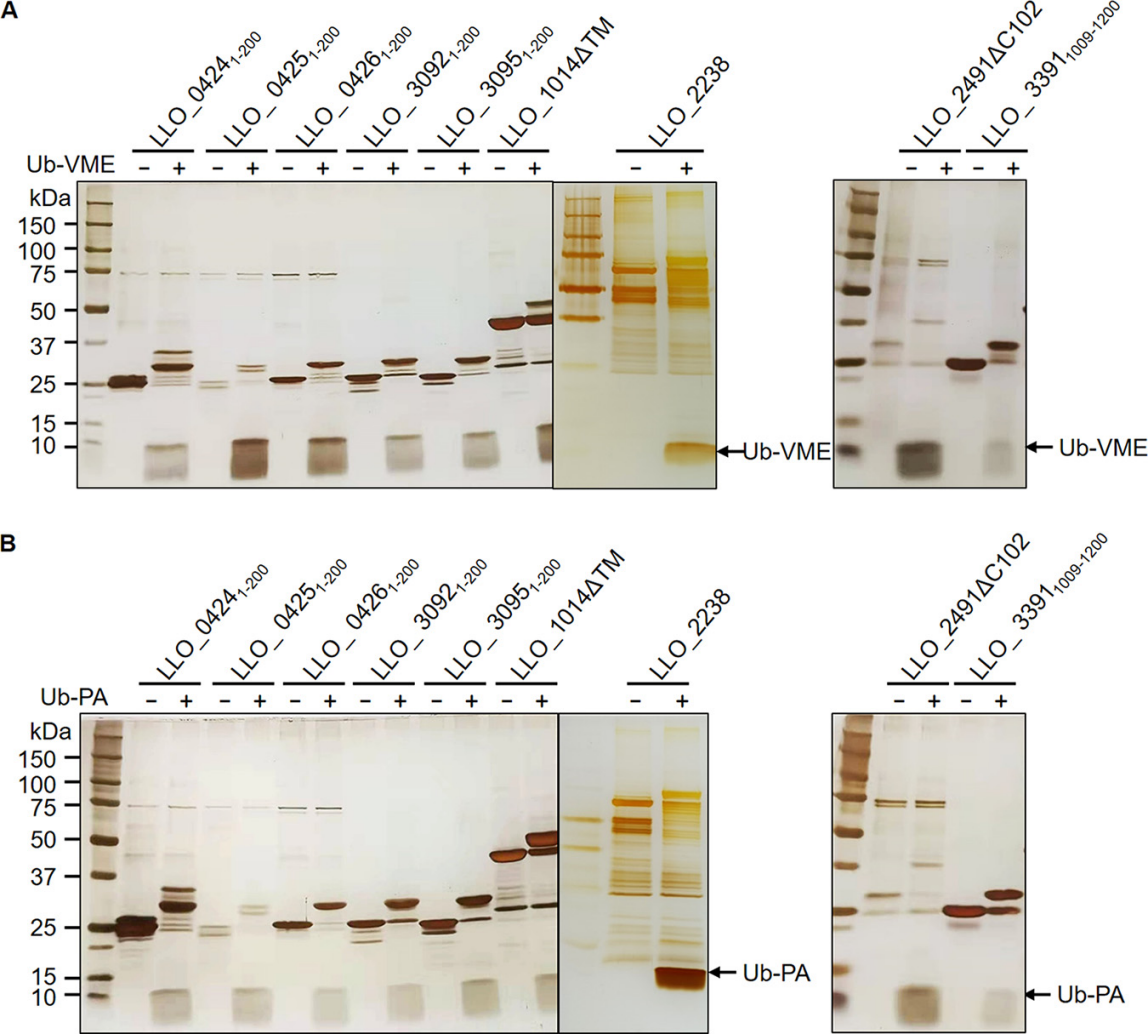
Reactivity of the potential DUBs with the suicide probes. The putative DUBs were incubated with Ub-VME (A) or Ub-PA (B) at 37°C for 2 h. Covalent modification of the DUBs by the suicide probes were detected by silver staining after SDS-PAGE. Results are one representative from three independent experiments.

**FIG 4:**
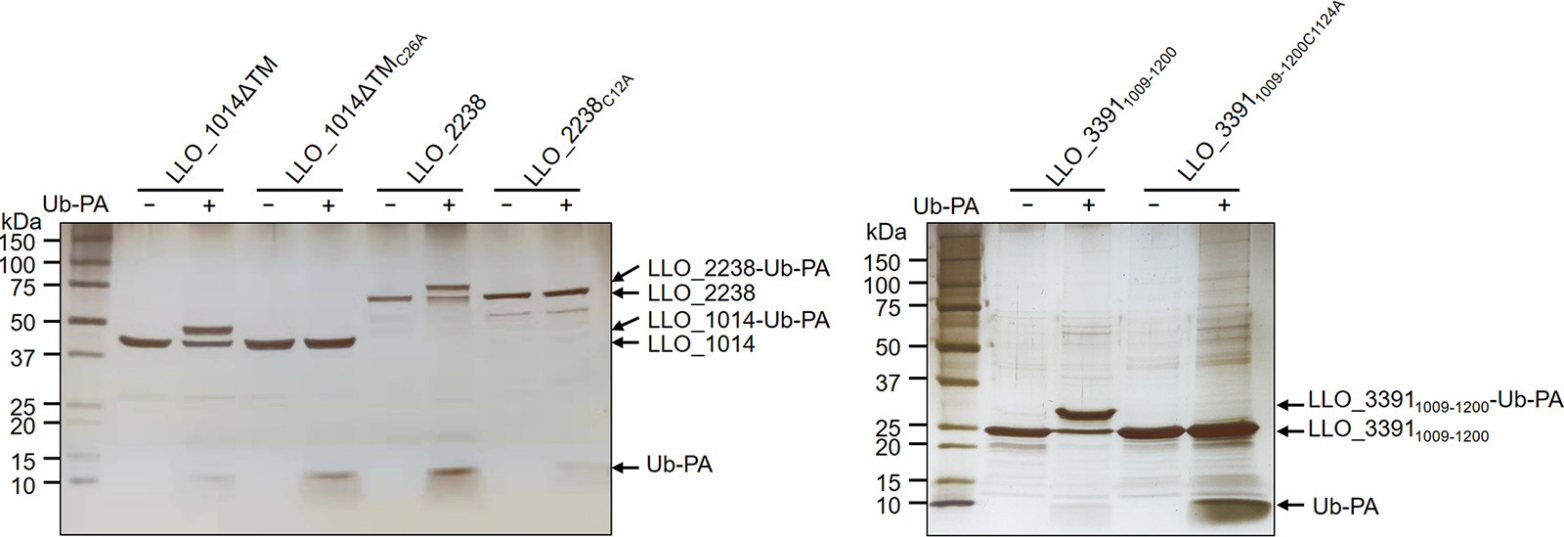
The reactivity of the potential DUBs with Ub-PA requires the catalytic cysteine. Recombinant proteins of the DUBs and their active cysteine substitution mutants were incubated with Ub-PA at 37°C for 2 h. After SDS-PAGE, proteins were detected by silver staining. Similar results are observed in three independent experiments.

To test whether the identified L. longbeachae proteins by our methods are bona fide DUBs, we first purified recombinant protein encoded by each of these genes from Escherichia coli. In a few cases, protein fragments harboring the predicted catalytic domains were constructed to circumvent the problem of expression and insolubility of full-length proteins (Table 3). Then, we examined their reactivity with Ub-VME and Ub-propargylamide (Ub-PA), two commonly used suicide probes for DUBs. Incubation of the DUB domains of SidE_LLO_ (LLO_3092, LLO_3095, LLO_0424, LLO_0425, LLO_0426), LLO_1014ΔTM, LLO_2238, and LLO_ 3391_1009–1200_ with both probes resulted in the production of covalent conjugates typical of active DUBs, which are featured by an ~8-kDa upshift in molecular weight (Fig. 3). In addition, mutation of the predicted catalytic cysteine residues in LLO_1014ΔTM, LLO_2238, and LLO_3391_1009–1200_ abolished such modification, indicative of the cysteine-based DUB activity of these proteins (Fig. 4). In contrast, we did not observe detectable reactivity with either Ub-VME or Ub-PA for LLO_0794, LLO_1369, LLO_1631, LLO_3118, LLO_2210, LLO_2179, LLO_2985, and LLO_2066 (Fig. S1).

**TABLE 3:** *In vitro* DUB activity of potential proteins

Protein	Purified fragment	Active cysteine	Reactivity with Ub-VME/Ub-PA	Cleavage of diubiquitin to:							
				K6	K11	K27	K29	K33	K48	K63	Linear
LLO_3092	1–200	Cys117	Both	Yes	Yes	No	No	Yes	Yes	Yes	No
LLO_3095	1–200	Cys117	Both	Yes	Yes	No	Yes	Yes	Yes	Yes	No
LLO_0424	1–200	Cys117	Both	Yes	Yes	No	No	Yes	Yes	Yes	No
LLO_0425	1–200	Cys117	Both	Yes	Yes	No	No	Yes	Yes	Yes	No
LLO_0426	1–200	Cys117	Both	Yes	Yes	No	No	Yes	Yes	Yes	No
LLO_0794	Full length	Unknown	No	No	No	No	No	No	No	No	No
LLO_1369	Full length	Unknown	No	No	No	No	No	No	Yes	Yes	No
LLO_1631	Full length	Unknown	No	No	No	No	No	No	No	No	No
LLO_3118	Full length	Unknown	No	No	No	No	No	No	No	No	No
LLO_2210	Full length	Unknown	No	No	No	No	No	No	No	No	No
LLO_2179	Full length	Unknown	No	No	No	No	No	No	No	No	No
LLO_2985	Full length	Unknown	No	No	No	No	No	No	No	No	No
LLO_1014	LLO_1014ΔTM	Cys26	Both	No	No	No	No	No	No	Yes	No
LLO_2238	Full length	Cys12	Both	Yes	Yes	Yes	Yes	Yes	Yes	Yes	No
LLO_2491	LLO_2491ΔC102	Cys13	Both	Yes	Yes	No	No	Yes	Yes	Yes	No
LLO_3391	1009−1200	Cys1124	Both	Yes	Yes	Yes	No	Yes	Yes	Yes	No
LLO_2066	Full length	Cys156	No	No	No	No	No	No	No	No	No

**FIG 5:**
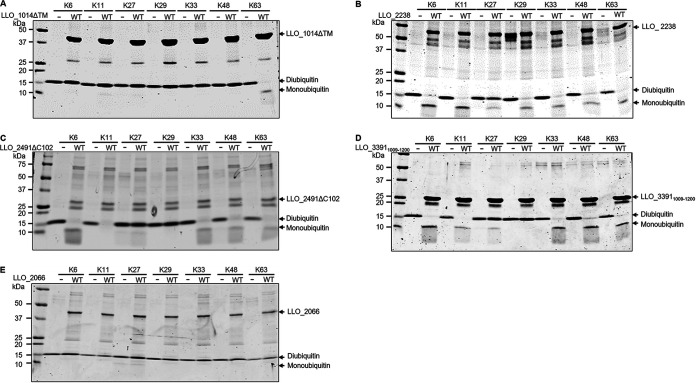
Cleavage of diubiquitin by putative L. longbeachae DUBs. A panel of diubiquitins with various linkage types was incubated with purified LLO_1014ΔTM (A), LLO_2238 (B), LLO_2491ΔC102 (C), LLO_3391_1009–1200_ (D), or LLO_2066 (E) at 37°C for 2 h. Protein samples separated by SDS-PAGE were detected by Coomassie brilliant blue (CBB) staining. Data shown are one representative from three independent experiments.

Next, we performed *in vitro* DUB assays using purified candidate proteins and a panel of diubiquitins formed by various linkage types. Consistently, candidate proteins that exhibited reactivity with ubiquitin suicide probes displayed DUB activity as evidenced by reduction in diubiquitin accompanied by an increase of monoubiquitin (Fig. 5 and Fig. S2). Six SidE_DUB_-containing proteins, including SidEs_LLO_ and LLO_3391_1009–1200_, efficiently hydrolyzed K6, K11, K33, K48, and K63 chains (Fig. 5, Fig. S2, and Table 3). In contrast, the three OTU domain-containing proteins possessed a clear preference for diubiquitins formed by different chain types (Fig. 5 and Table 3). LLO_1014 specifically processed the diubiquitin chain assembled by K63, whereas LLO_2238 showed activity against each of the lysine-linked diubiquitins (Fig. 5 and Table 3). As expected, the active cysteine mutants of these proteins have lost the ability to cleave diubiquitins (Fig. S3). On the contrary, LLO_2066 did not show catalytic activity to any of the diubiquitins (Fig. 5 and Table 3). Taken together, our data reveal that L. longbeachae encodes at least 9 proteins possessing DUB activity.

**FIG 6:**
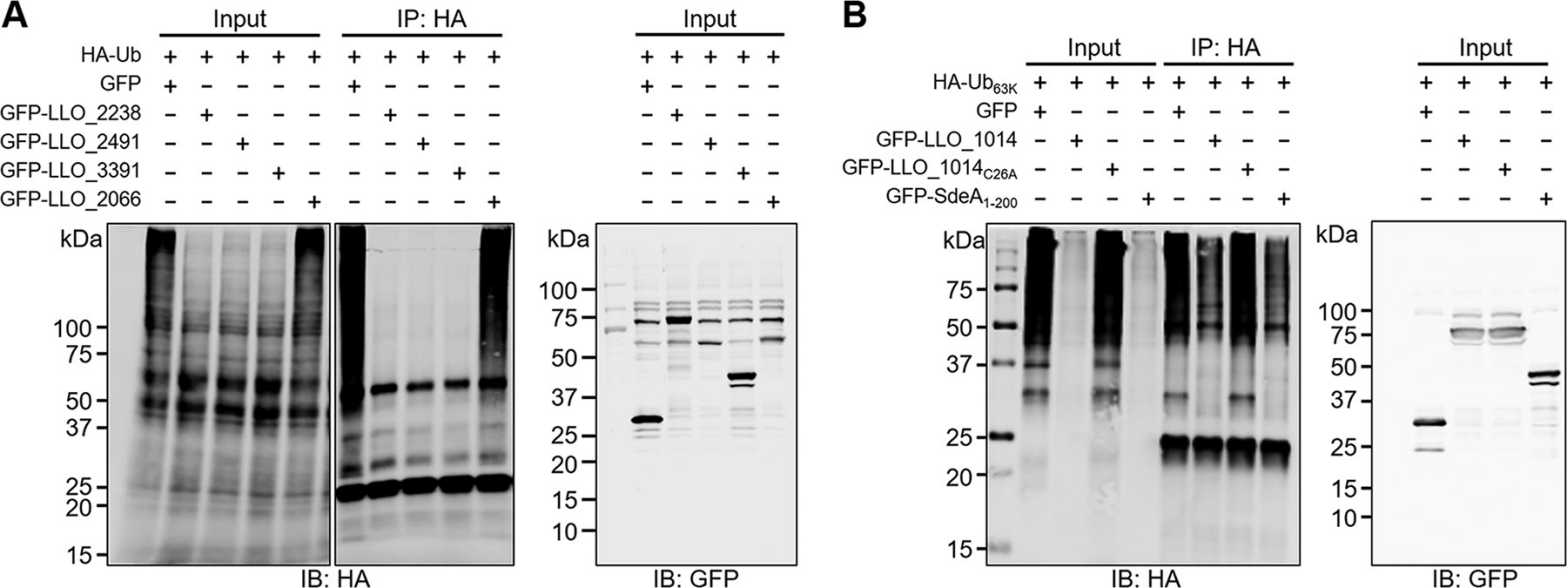
L. longbeachae DUBs interfere with protein ubiquitination in mammalian cells. (A) HEK293T cells were cotransfected with a plasmid producing HA-Ub and plasmids encoding GFP-LLO_2238, GFP-LLO_2491, GFP-LLO_3391_1009–1200_, or GFP-LLO_2066. Protein ubiquitination by HA-Ub was enriched by immunoprecipitation with anti-HA agarose and detected by Western blotting with the HA-specific antibody. The cell lysates were probed with a GFP-specific antibody to detect the expression of GFP fusion proteins. (B) HEK293T cells were transfected to coexpress HA-Ub_63K_ and GFP-LLO_1014 or GFP-LLO_1014_C26A_. Cellular proteins modified by K63-linked polyubiquitination were immunoprecipitated with the anti-HA agarose and evaluated by Western blotting with the anti-HA antibody. Expression of GFP-LLO_1014 and GFP-LLO_1014_C26A_ was detected by an anti-GFP antibody. GFP-SdeA_DUB_ was included as a positive DUB control. Data shown in panels A and B are one representative from three independent experiments.

To further characterize biochemically validated DUBs in L. longbeachae, we cotransfected HEK293T cells with HA-tagged ubiquitin and green fluorescent protein (GFP) fusions of LLO_2238, LLO_2491, LLO_3391, and LLO_2066. The cellular polyubiquitinated proteins enriched by the anti-HA agarose were detected by the HA-specific antibody. In agreement with the activity seen in our biochemical assays, ectopically expressed LLO_2238, LLO_2491, and LLO_3391, but not LLO_2066, markedly reduced cellular polyubiquitin levels (Fig. 6A). Considering its K63 linkage specificity, GFP-LLO_1014 was coexpressed with HA-Ub_63K_, a ubiquitin derivative that harbors only K63. Similarly, GFP-LLO_1014 decreases cellular proteins modified by K63-linked polyubiquitin chains in a way dependent on the catalytic cysteine (Fig. 6B).

### Two L. longbeachae DUBs are associated with the LCV after being injected into host cells.

**FIG 7:**
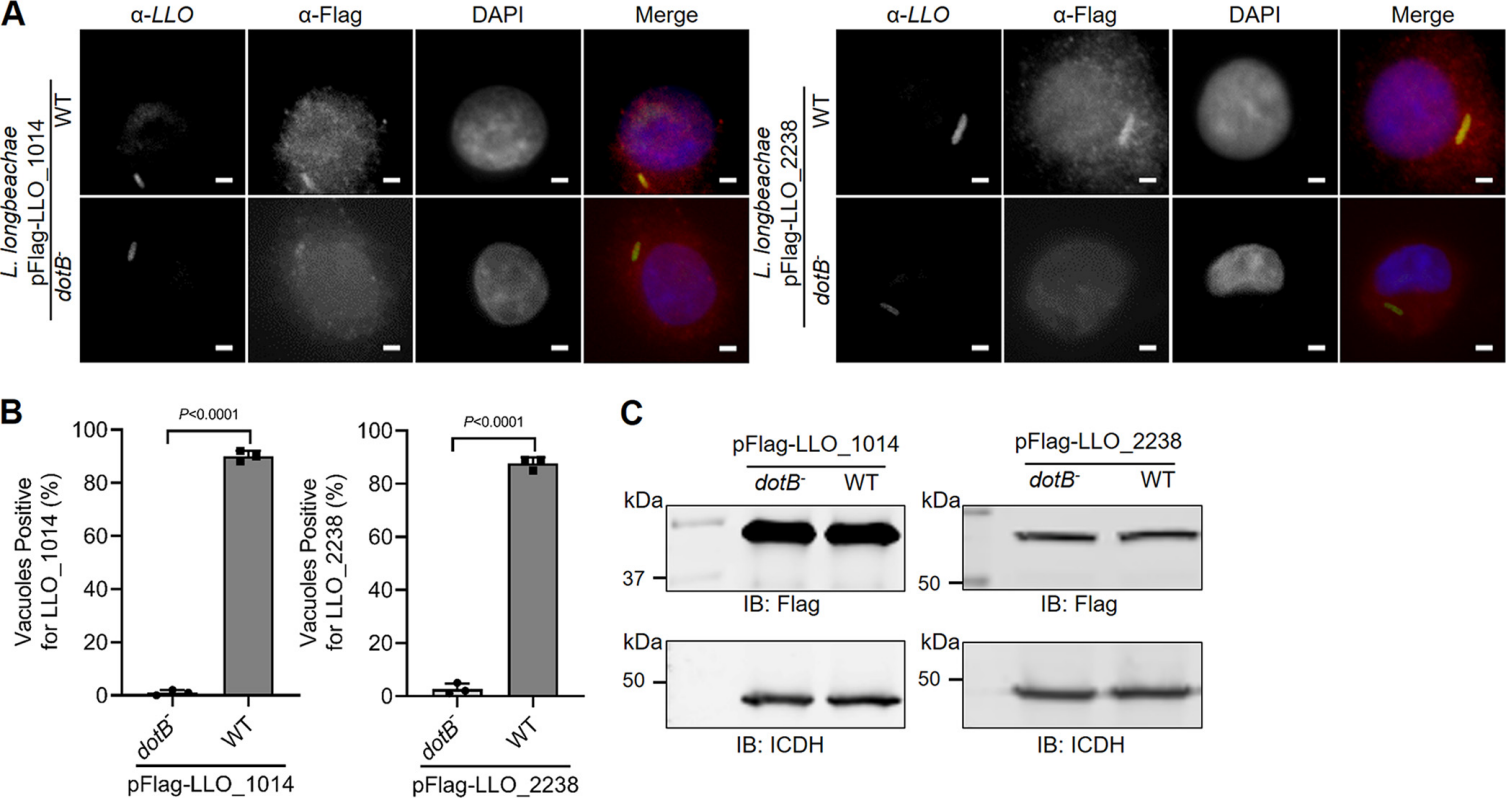
Association of L. longbeachae OTU family DUBs with LCV. (A) Wild-type and the Δ*dotB* mutant L. longbeachae strains were transformed with pXDC61JQ constructs to express LLO_1014 or LLO_2238 with an N-terminal Flag tag. U937 cells infected for 2 h with these strains were fixed and subsequently immunostained with anti-L. longbeachae and anti-Flag antibodies. The nuclei were stained with DAPI (dark blue). Representative images were acquired by a fluorescence microscope. Scale bar, 2 μm. (B) Percentage of LCVs positive for Flag staining signals. At least 100 bacterial vacuoles were examined for each sample. (C) The expression of the Flag fusion proteins in L. longbeachae strains was detected by Western blotting with an anti-Flag antibody. The isocitrate dehydrogenase (ICDH) was probed as a loading control. Data shown in panel B are mean ± SD scored from three coverslips. Panels B and C are one representative of three independent assays.

Since the ubiquitin system exclusively exists in eukaryotic organisms, the recognition and delivery by specific bacterial secretion systems into host cells is the prerequisite for bacterial DUBs to execute their regulatory functions. Having proven the DUB activity in biochemical reactions and ectopically expressed mammalian cells, we next investigated whether the identified DUBs are the Dot/Icm substrate of L. longbeachae. To this end, we constructed plasmids that directed the expression of Flag-tagged LLO_1014 and LLO_2238 and introduced them into both WT L. longbeachae and a *dotB* deletion mutant, respectively. Then, U937 cells were infected with these strains and subjected to stepwise immunostaining with antibodies specifically recognizing L. longbeachae and the Flag epitope. Clear Flag staining signals were detected on the LCVs (Fig. 7A). Approximately 90% and 87% of bacterial phagosomes stained positive for Flag in cells challenged with WT L. longbeachae expressing Flag-LLO_1014 and LLO_2238, respectively (Fig. 7A and B). Such association is strictly dependent on a functional Dot/Icm system. Despite producing a similar amount of the fusion proteins, less than 2% of the vacuoles harboring the Δ*dotB* strains stained positive for Flag (Fig. 7A and B). Hence, both LLO_1014 and LLO_2238 are L. longbeachae Dot/Icm substrates, which are localized to the LCV membrane after being translocated by the Dot/Icm system.

### L. longbeachae DUBs regulate the association of polyubiquitinated proteins on the LCV.

**FIG 8:**
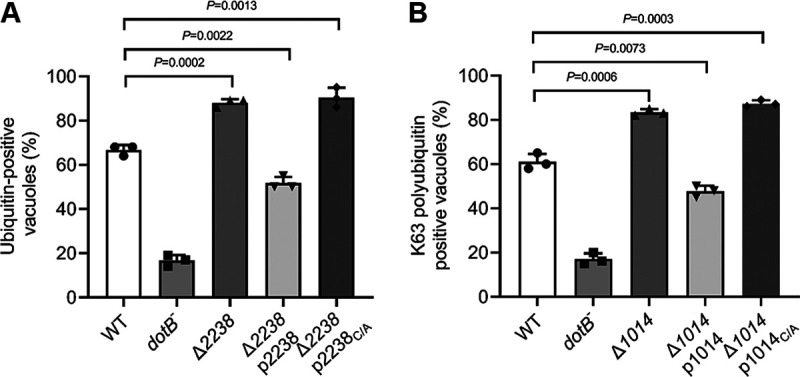
L. longbeachae OTU family DUBs regulate the association of polyubiquitinated proteins with their phagosome. (A and B) U937 cells infected with indicated L. longbeachae strains for 2 h (MOI = 10) were fixed infection samples and were stained with L. longbeachae and FK1 (A) or K63-linkage-specific (B) polyubiquitin antibodies to detect ubiquitinated proteins on the bacterial phagosome. The proportion of LCVs positively stained with ubiquitin antibodies is shown. At least 100 phagosomes were counted for each sample, and data are presented as mean ± SD scored from three coverslips. Similar results are obtained from three independent experiments. Statistics analysis was conducted via unpaired two-tailed Student *t* tests, and a *P* value of <0.05 indicates a significant difference.

LCVs harboring L. longbeachae are highly enriched with polyubiquitinated proteins in a Dot/Icm-dependent manner, suggesting the involvement of effector proteins in such decoration. The association of DUBs with the LCV harboring L. longbeachae prompts us to assess whether these enzymes impact the accumulation of polyubiquitinated species on the bacterial phagosome. To test this, we constructed L. longbeachae deletion mutants lacking individual DUB genes. After infection of U937 cells with relevant L. longbeachae strains for 2 h, the percentage of ubiquitin-decorated vacuoles was probed by ubiquitin-specific antibodies and evaluated under a fluorescence microscope. Compared to samples infected with wild-type L. longbeachae, LCVs that stained positive for polyubiquitin increased to 88% in samples infected with the Δ*LLO_2238* mutant (Fig. 8A). Similarly, deletion of LLO_1014 resulted in a significant increase in the percentage of LCVs that stained positive for K63-linked polyubiquitin (Fig. 8B). Importantly, for each tested DUB mutant, we observed complementation of LCV polyubiquitination by plasmids carrying wild-type DUB genes but not their catalytic cysteine mutants (Fig. 8A and B). Together, these data suggest that LLO_1014 and LLO_2238 act to remove polyubiquitin on the bacterial phagosome.

### Two OTU DUBs from L. longbeachae and L. pneumophila are functionally exchangeable.

The L. longbeachae OTU-containing proteins LLO_1014 and LLO_2238 share 60.4% and 54.3% overall identity with their L. pneumophila orthologous Ceg23 (LotB) and Lem27 (LotC), respectively (Fig. S4). More importantly, despite the differences in primary sequences, the DUB activity, as well as the chain preferences, are highly conserved between the ortholog proteins. Both LLO_1014 and Ceg23 exclusively process K63-linked ubiquitin chains, whereas LLO_2238 and Lem27 possess DUB activity against each of the lysine-linked ubiquitin chains. These observations prompted us to examine the potential functional equivalent of these proteins. To this end, we first employed a β-lactamase (TEM) reporter assay to characterize whether LLO_1014 and LLO_2238 can be translocated by the Dot/Icm transporter of L. pneumophila. Approximately 54% and 51% of infection of Raw264.7 cells with L. pneumophila strains expressing TEM-LLO_1014 and TEM-LLO_2238 led to translocation of the fusion proteins, as evidenced by emission of blue fluorescence signals (Fig. S5). As controls, translocation did not occur in cells infected with L. pneumophila Δ*dotA* strains producing a similar amount of the fusion proteins (Fig. S5).

**FIG 9:**
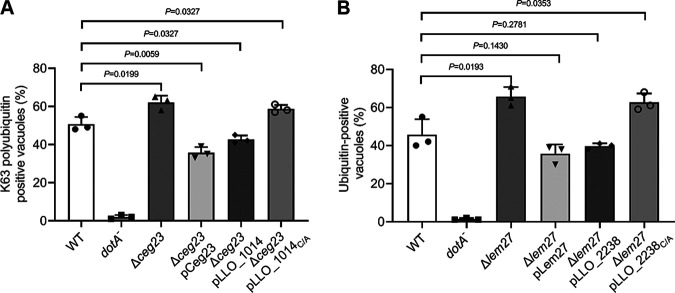
Complementation of phenotypes associated with L. pneumophila OTU mutants with counterparts from L. longbeachae. (A and B) Plasmids coding for the relevant L. longbeachae OTU genes and their derivatives were transformed into L. pneumophila Δ*lem27* or Δ*ceg23*. U937 cells were challenged with indicated L. pneumophila strains for 2 h at an MOI of 10. Cells were then fixed and sequentially immunostained with L. pneumophila and K63-linkage specific (A) or FK1 (B) polyubiquitin antibodies. Staining signals were visualized by a fluorescence microscope. The percentage of phagosomes decorated with polyubiquitinated proteins. Results are mean ± SD counted from three coverslips. Similar results are observed in three independent experiments. Statistics analysis was performed by unpaired two-tailed Student *t* tests, and a *P* value of <0.05 indicates a significant difference.

Next, we measured the ubiquitination status of LCVs in cells challenged with relevant L. pneumophila strains. Fifty percent of the phagosomes harboring WT L. pneumophila were enriched with K63-linked polyubiquitin. Consistent with our previous findings, deletion of *ceg23* significantly promoted K63-linked polyubiquitin on vacuoles (35), as 62% of LCVs stained positive by the K63-polyubiquitin-specific antibody (Fig. 9A). Importantly, this phenotype can be reversed by the expression of either Ceg23 or its L. longbeachae ortholog, LLO_1014 (Fig. 9A). Similarly, the elevation of polyubiquitin-decorated LCVs, due to the lack of *lem27* (36), can be fully complemented by plasmids expressing *lem27* or its L. longbeachae ortholog *llo_2238* (Fig. 9B). Furthermore, although LLO_1014_C/A_ and LLO_2238_C/A_ were similarly expressed in the relevant mutant strains, the percentage of ubiquitin-enriched vacuoles cannot be restored to the levels displayed by the wild-type strain (Fig. 9A and B). Taken together, our results suggest that OTU family DUBs in L. longbeachae and L. pneumophila are functionally exchangeable in regulating the association of polyubiquitin on the LCV.

### L. longbeachae DUBs are dispensable for bacterial intracellular growth in macrophages.

To test whether the identified DUBs are required for L. longbeachae intracellular replication, we infected U937 cells with L. longbeachae strains lacking individual DUB genes. The WT L. longbeachae strain grew approximately 200-fold in 72 h (Fig. S6). In contrast, no growth was observed for the *dotB* deletion strain (Fig. S6). Notably, L. longbeachae lacking *LLO_1014*, *LLO_2238*, *LLO_2491*, or *LLO_3391* grew indistinguishably from that of the wild-type strain (Fig. S6), suggesting that these DUBs are dispensable for L. longbeachae growth in U937 cells.

## DISCUSSION

Since the discovery of L. pneumophila in 1976, over 65 *Legionella* species have been identified, and half of them are associated with Legionnaires’ disease (17). The molecular pathogenesis research of *Legionella* has been predominantly studied for L. pneumophila in the past decades. Yet, as the second leading cause of Legionnaires’ disease, little is known about L. longbeachae’s biology and infection process. Importantly, a recent study demonstrated that L. longbeachae is more virulent than L. pneumophila in a mouse model of infection (37). Therefore, along with the increasingly reported cases of L. longbeachae infections (19), it is necessary to investigate the molecular pathogenesis of this bacterium.

Despite causing clinically indistinguishable diseases, L. longbeachae and L. pneumophila differ in their environmental niches, physiologies, and transmission routes (20). Particularly, although the Dot/Icm machinery is highly conserved, the effector repertoires of the two *Legionella* species display considerable variations (20, 24, 29). Such genotypic differences are supposed to be manifested in its interactions with hosts. In this study, we demonstrate that the phagosome harboring virulent L. longbeachae is extensively decorated with polyubiquitin species, and a functional ubiquitin system is required for its optimal intracellular growth. Through systematic identification and validation, we found a total of 9 L. longbeachae DUBs that may potentially participate in the interference of host ubiquitin network. Moreover, we proved that several LCV-associated DUBs are critical for remodeling the LCV by polyubiquitination.

The extensive association of polyubiquitinated proteins with the LCV is a critical feature of bacterial phagosome formed by virulent L. pneumophila (26). Although the exact roles of such recruitment are still not fully understood, one possible function is to facilitate protein degradation, thus providing amino acids required for bacterial proliferation. Indeed, suppression of the host ubiquitin system by MG-132 or silencing of the AAA+ ATPase Cdc48/p97 impairs intracellular L. pneumophila growth (26), further highlighting the importance of the ubiquitin network in L. pneumophila virulence. Recently, it was demonstrated that the characteristics of LCVs bearing L. longbeachae resemble those formed by L. pneumophila in such aspects as the recruitment of calnexin, Rab1, and Sec22b (30). In line with these findings, our study revealed the similar ubiquitin-decorated bacterial phagosome of L. longbeachae as L. pneumophila, further supporting the theory that both *Legionella* species develop phenotypically similar replicative vacuoles, albeit likely through distinct mechanisms. On one hand, the variations in the effector protein sets among *Legionella* species suggest that they may utilize distinct virulence determinants to achieve the same goal. Indeed, L. pneumophila Rab1-modifying effectors such as SidM/DrrA, AnkX, Lem3, and SidD are not encoded by L. longbeachae, indicative of a novel mechanism to recruit Rab1 to the phagosome. Intriguingly, L. longbeachae translocates a unique family of Rab GTPase-like effectors that may provide an alternative means to recruit ER-derived vesicles to remodel its LCV (30). On the other hand, L. pneumophila and L. longbeachae appear to share common mechanisms in the endoplasmic reticulum (ER) recruitment, which both require the PI4P-binding effector protein SidC (38).

To date, a total of 12 L. pneumophila effectors have been shown to act as DUBs that are grouped into different subfamilies (39). Once translocated into host cells, these proteins extensively interfere with host ubiquitin-related signaling events. Importantly, L. pneumophila encodes RavD, which specifically hydrolyzes linear ubiquitin chains, thereby inhibiting host inflammatory signaling (40). Interestingly, this phenotype is unique to L. pneumophila, as most *Legionella* species, including L. longbeachae, lack a RavD ortholog (24, 40). Alternatively, these species may target linear ubiquitin chains by utilizing an enzyme which is structurally different from RavD.

In this study, we systematically profiled the DUB proteins encoded by L. longbeachae. The SidEs DUB module of L. pneumophila was found in six L. longbeachae proteins, including five SidE orthologs. In addition, except for LotA, a DUB of the OTU family that favors the K6 linkage type (41), all other L. pneumophila OTU DUBs are present in L. longbeachae (35, 36, 42–44). Importantly, despite the significant variations in primary sequences, the ubiquitin chain preferences of the orthologous DUBs are highly conserved. For example, both LLO_1014 and its L. pneumophila ortholog Ceg23 are K63-specific DUBs (35, 42). The OTU family DUBs in L. longbeachae and L. pneumophila seem to be functionally equivalent, as evidenced by the ability of LLO_1014 and LLO_2238 to fully reduce the increased ubiquitin association on the LCV displayed by the Δ*ceg23* and Δ*lem27* mutants of L. pneumophila, respectively (35, 36).

In addition to DUBs, L. pneumophila codes for a large number of ubiquitin E3 ligases that employ distinct catalytic mechanisms (25). Importantly, these E3s and DUBs appear to act in concert to maintain the homeostasis of ubiquitinated proteins on the LCV. For example, L. pneumophila DUB Lem27 (LotC) appears to reverse the SidC-induced Rab10 ubiquitination and reduce its association with the phagosome (36). Considering the redundancy of the OTU DUBs in the regulation of LCV polyubiquitination, it is reasonable to speculate that such interplay between E3 ligases and DUBs may also exist during L. longbeachae infection. In fact, although L. longbeachae SidC only shares 40% identity with its L. pneumophila ortholog, the catalytic Cys-His-Asp motif required for its E3 ligase activity is conserved (45). Hence, investigation of E3 ligases coded for by L. longbeachae will provide a better understanding of the mechanisms used by this bacterium to coopt the host ubiquitin network. Furthermore, future studies aiming to identify host substrates targeted by L. longbeachae DUBs are necessary to clarify how these DUBs impact ubiquitin signaling to promote bacterial survival and intracellular growth.

## MATERIALS AND METHODS

### Strains, plasmids, and culture methods.

Bacterial strains, plasmids, and primers used in this study are shown in Tables S1 to S3 in the supplemental material, respectively. Escherichia coli strains DH5αλπ and BL21(DE3) were cultured in LB medium at 37°C. When necessary, antibiotics were supplemented to the E. coli cultures at the following concentrations: 100 μg/mL ampicillin, 30 μg/mL kanamycin, and 30 μg/mL chloramphenicol. L. pneumophila Philadelphia 1 strain Lp02, L. longbeachae ATCC 33462, and derivatives of these strains were grown at 37°C on charcoal-yeast extract (CYE) plates or in ACES [*N*-(2-acetamido)-2-aminoethanesulfonic acid]-buffered yeast extract (AYE) broth. If needed, *Legionella* cultures were supplemented with appropriate antibiotics at the following concentrations: 20 μg/mL kanamycin, 50 μg/mL streptomycin, and 5 μg/mL chloramphenicol. In-frame deletion mutants of L. longbeachae were constructed as previously described methods (46). In brief, the up- and downstream flanking regions of the target gene were amplified from the genomic DNA of L. longbeachae. After digesting with appropriate enzymes, the fragments were inserted into pSR47s by three-way ligation. The resulting plasmids were introduced into L. longbeachae by electroporation and plated on CYE agar plates containing kanamycin and streptomycin. The resulting colonies were then plated onto CYE agar supplemented with 5% sucrose and cultured for 4 days. Finally, successful deletion of the target gene was screened by PCR. For translocation of proteins by L. longbeachae, PCR products were inserted into pXDC61m (47).

To ectopically express proteins in mammalian cells and E. coli, genes of interest were inserted into peGFPC1 and pET28a, respectively. To measure translocation of proteins by L. pneumophila, PCR products were inserted into pXDC61m. For complementation of L. pneumophila, amplified DNA products were cloned into pZL507 (48) and electroporated into the host strains. The pXDC61m-derived plasmid pXDC61JQ was used to produce Flag-tagged proteins in the L. longbeachae. To construct pXDC61JQ, a double-stranded DNA fragment with sticky ends (NdeΙ-Flag-BamHΙ-BglΙΙ-SacΙ-XhoΙ-SalΙ-HindΙΙΙ) obtained by annealing oligonucleotides was inserted into NdeΙ/HindIII-digested pXDC61m (47). Site-directed mutagenesis of the genes was performed by the QuikChange kit (Agilent Technologies) and verified by DNA sequencing.

### Cell lines and transfection.

HEK293T cells were cultured in Dulbecco’s modified minimal Eagle’s medium (HyClone) supplemented with 10% (vol/vol) fetal bovine serum (FBS). RAW264.7 and U937 cells were cultured in RPMI medium supplemented with 10% FBS. U937 cells were differentiated into macrophages by phorbol-12-myristate-13-acetate (PMA) prior to bacterial infection. All cell lines were grown in a 5% CO_2_ incubator at 37°C. Transfection of HEK293T cells was performed with Lipofectamine 3000 (Invitrogen) according to the manufacturer’s protocol.

### Identification of potential L. longbeachae DUBs.

L. longbeachae was grown in 1 L of AYE broth to the postexponential phase (optical density at 600 nm [OD_600_] = 3.3 to 3.8). Cells harvested by centrifugation at 5,000 × *g* for 20 min were resuspended in 30 mL of the lysis buffer (300 mM NaCl, 20 mM Tris-HCl, pH 7.5). After lysis by high-pressure homogenization (JN-mini; JNBIO, Guangzhou, China), the soluble fractions were collected by centrifugation at 20,000 × *g* for 1 h at 4°C. To capture potential DUBs in L. longbeachae by the activity-based probe, 1 μM HA-Ub-VME was incubated with 1 mL of L. longbeachae lysates at 37°C for 2 h. Then, the probe reactive proteins were enriched by immunoprecipitation with the anti-HA agarose (Sigma) and analyzed by mass spectrometry (Applied Protein Technology Biotech, Shanghai, China).

A total of 992 hypothetical proteins were retrieved from the L. longbeachae genome sequence (NCBI reference sequence, GenBank accession no. NC_013861). For bioinformatics analysis, amino acid sequences of the candidates were analyzed via the HHpred server using default parameters (https://toolkit.tuebingen.mpg.de/#/tools/hhpred) (34).

### Recombinant protein purification.

E. coli BL21(DE3) carrying desired pET28a-derived plasmids was cultured at 37°C in LB broth supplemented with kanamycin (30 μg/mL). When the OD_600_ reached approximately 0.6 to 0.8, recombinant protein expression was induced by 0.2 mM isopropyl β-d-1-thiogalactopyranoside (IPTG) and further incubated for 16 h at 18°C with constant shaking (220 rpm). Cells pelleted by centrifugation were resuspended in a lysis buffer (50 mM NaH_2_PO_4_, 300 mM NaCl, and 10 mM imidazole, pH 8.0) and lysed via a high-pressure homogenization (JN-mini). After removing the cell debris by spinning at 20,000 × *g* for 30 min, the supernatants were incubated with 1 mL of prewashed Ni^2+^-nitrilotriacetic acid (NTA) beads (Qiagen) for 2 h at 4°C in a head-to-end rotator. Unbound proteins were cleared by the washing buffer (50 mM NaH_2_PO_4_, 300 mM NaCl, and 20 mM imidazole, pH 8.0). Then, the His_6_-tagged proteins were eluted by the elution buffer (50 mM NaH_2_PO_4_, 300 mM NaCl, and 250 mM imidazole, pH 8.0). The resulting proteins were dialyzed twice in a buffer containing 300 mM NaCl, 20 mM Tris-HCl (pH 7.5), and 10% glycerol. Protein concentrations were measured by the Bradford protein assay (Bio-Rad).

### Deubiquitination assays.

To detect the reactivity of the potential DUBs with the suicide probes, 1 μM Ub-PA or Ub-VME (Boston Biochem) was mixed with 1 μM His_6_-tagged proteins in 20 μL DUB buffer (50 mM Tris-HCl, pH 7.5, 50 mM NaCl, and 2 mM dithiothreitol [DTT]) and incubated at 37°C for 1 h. Reactions were terminated by the addition of 5 μL 5× SDS loading buffer and boiling for 5 min at 95°C. The formation of ubiquitin adducts was visualized by silver staining.

For *in vitro* cleavage of diubiquitins, 1 μM of the potential DUBs was reacted with 1 μM each with diubiquitin in 20 μL DUB buffer (500 mM Tris-HCl, pH 7.5, 50 mM NaCl, and 2 mM DTT) for 2 h at 37°C. We added 5× SDS loading buffer to the mixtures to stop the reactions. Samples separated by SDS-PAGE were further visualized by Coomassie brilliant blue staining.

To determine the DUB activity in mammalian cells, a plasmid driving the expression of HA-Ub (33) or HA-Ub_63K_ (35) was cotransfected into HEK293T cells with constructs expressing GFP-tagged DUB proteins. Twenty-four hours posttransfection, cells were lysed by NP-40 lysis buffer for 20 min on ice. After removing the insoluble fractions by centrifugation at 12,000 × *g* for 10 min at 4°C, the supernatants were subjected to immunoprecipitation with anti-Flag or anti-HA agaroses for 4 h with constant rotation. Polyubiquitin species retained on the beads were detected by immunoblotting with appropriate primary antibodies.

### Bacterial infections and immunostaining.

For infection experiments, L. pneumophila and L. longbeachae strains were cultured in AYE broth at 37°C to postexponential phase (OD_600_ = 3.3 to 3.8). When necessary, *Legionella* cultures were supplemented with 0.5 mM IPTG to induce protein expression.

For intracellular growth assays, 4 × 10^5^/well U937-derived macrophages were seeded into 24-well plates and infected with relevant L. longbeachae strains (MOI = 10). At 2 h of infection, the cells were washed with prewarmed phosphate-buffered saline (PBS) to remove extracellular bacteria and supplemented with fresh media. The cells were lysed with 0.02% saponin at 2, 24, 48, and 72 h postinfection. Diluted lysates were plated on CYE plates and grown at 37°C until the emergence of obvious colonies. CFU were calculated from infections done in triplicate for each strain to evaluate intracellular bacterial growth.

To determine the importance of host proteasome on the intracellular replication of *Legionella*, U937 cells were pretreated with indicated concentrations of MG-132 for 1 h before infection. After removing the inhibitor by washing the wells with prewarmed PBS, the cells were infected with relevant *Legionella* strains at a multiplicity of infection (MOI) of 10 for 2 h. Samples were washed with PBS to remove extracellular bacteria and supplemented with fresh media. At 14 h postinfection, cells were fixed and stained by anti-*Legionella* antibodies following the subsequently described immunostaining procedures.

To determine the association of effector proteins and ubiquitinated proteins on the LCV, U937 cells (5 × 10^4^/well) seeded on coverslips in 24-well plates were infected with relevant *Legionella* species strains (MOI = 10) for 2 h. Infection samples were fixed by 4% paraformaldehyde for 15 min at room temperature (RT). The extracellular bacteria were stained with anti-*Legionella* antibodies produced in rat prior to being permeabilized by 0.2% Triton X-100 at room temperature for 5 min. Then, cells were blocked with 4% goat serum in PBS for 30 min at RT followed by immunostaining with primary antibodies. Anti-L. longbeachae and anti-L. pneumophila polyclonal antibodies produced in rat and rabbit were commercially generated by AbMax Biotechnology Co., Ltd. (Beijing, China) and diluted at 1:1,000 and 1:2,000 for immunostaining, respectively. Other antibodies used in this study are as follows: rabbit anti-L. pneumophila (1:10,000) (35), rat anti-L. pneumophila (1:2,000) (28), anti-Flag (Sigma; catalog no. F1804; 1:200); anti-FK1 (Enzo; product no. BML-PW8805; 1:1,000); and Lys-63-specific ubiquitin chains (EMD Millipore; catalog no. 05-1308; 1:50). After incubation with appropriate fluorescence dye-conjugated secondary antibodies for 1 h at RT, the staining signals were visualized using an Olympus IX-83 fluorescence microscope. All image analyses were conducted blind by coding the coverslips before experiments.

For translocation assay, Raw264.7 cells were infected with WT and *dotA*^−^ mutant L. pneumophila strains producing TEM fusion proteins at an MOI of 50 for 2 h. After washing the infection samples with PBS 3 times, 20 μL of 6× CCF4-AM solution (LiveBLAzer-FRET B/G loading kit; Invitrogen) was added to the cells and incubated in dark for 2 h. Protein translocation by L. pneumophila was inspected by a fluorescence microscope (IX83; Olympus) equipped with a β-lactamase FL-Cube (U-N41031; Chroma Technology Corp., Bellows Falls, VT).

### Western blot analysis.

For Western blot analysis, samples separated by SDS-PAGE were then transferred to nitrocellulose membranes (Pall Life Sciences). Primary antibodies used are as follows: anti-Flag (Sigma; catalog no. F1804; 1:3,000), anti-GFP (Proteintech; catalog no. 50430-2-AP; 1:5,000), anti-isocitrate dehydrogenase (ICDH) (1:10,000) (49), anti-TEM (Abcam; catalog no. ab12251; 1:3,000), anti-LLO_2238 (1:2,000), and anti-LLO_1014 (1:2,000). Rabbit polyclonal antibodies against LLO_1014 and LLO_2238 were produced by immunization of rabbits with recombinant His_6_-LLO_1014ΔTM and His_6_-LLO_2238 (AbMax Biotechnology Co., Ltd., Beijing, China). After incubation of the membranes with appropriate IRDye-conjugated secondary antibodies (Li-Cor), the signals were detected by an Odyssey CLx imaging system (Li-Cor).

### Data analysis.

The unpaired two-tailed Student *t* tests were used to analyze the data, and a *P* value of <0.05 was considered a significant difference.
