# Supplementary material for: Legionella longbeachae Regulates the Association of Polyubiquitinated Proteins on Bacterial Phagosome with Multiple Deubiquitinases
Source: Microbiol Spectr. 2023 Feb 15;11(2):e04179-22. doi: 10.1128/spectrum.04179-22 (PMC10100730; doi:10.1128/spectrum.04179-22)
Supplement: Supplemental file 1 — Supplemental material. Download spectrum.04179-22-s0001.pdf, PDF file, 2.3 MB [file spectrum.04179-22-s0001.pdf]

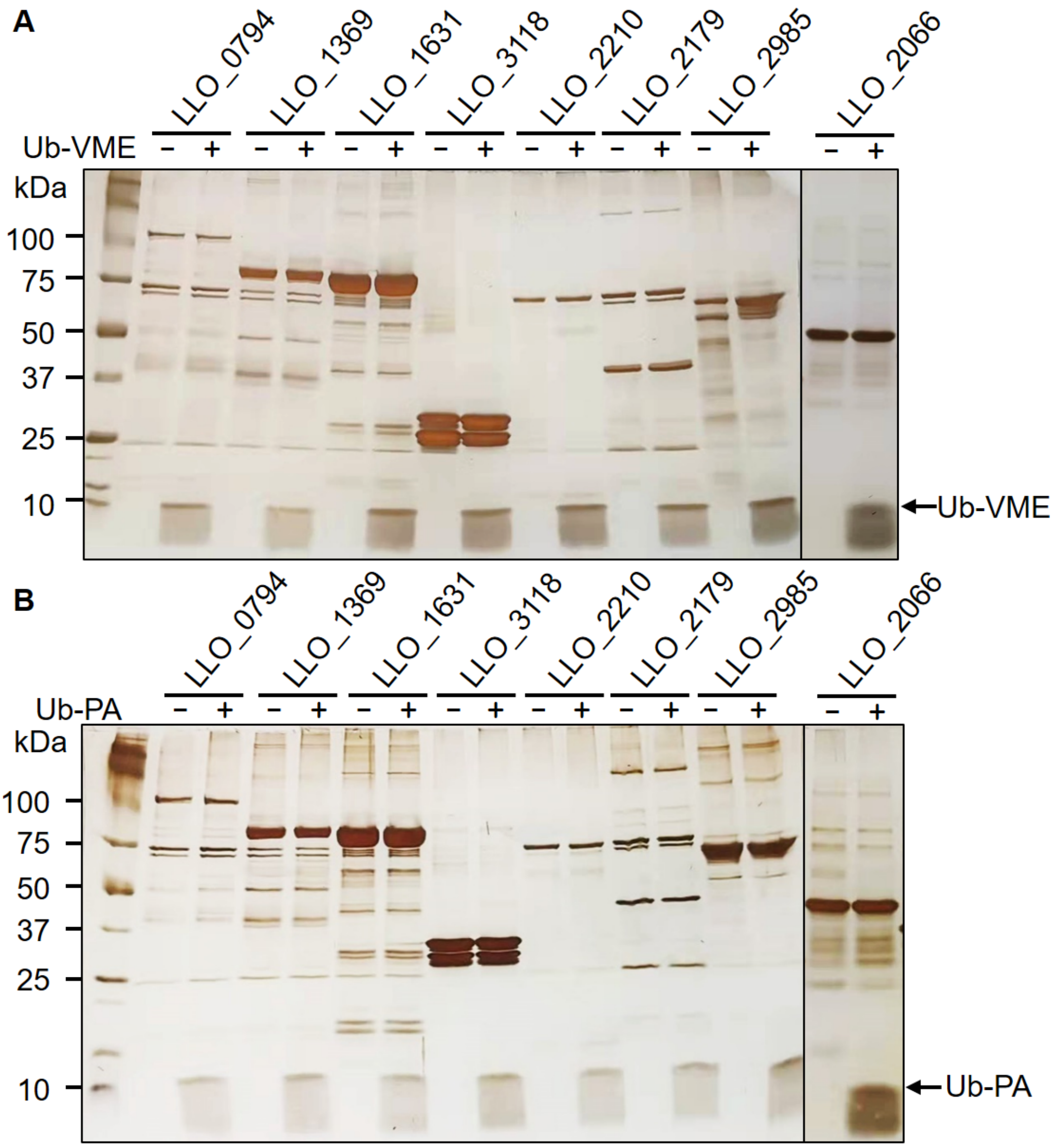

FIG S1. Reactivity of the candidate proteins with the suicide probes. Recombinant proteins purified from *E. coli* were reacted with Ub-VME (A) or Ub-PA (B) at 37 °C for 2 h. Proteins separated by SDS-PAGE were subjected to silver staining. Similar results are observed in three independent experiments.

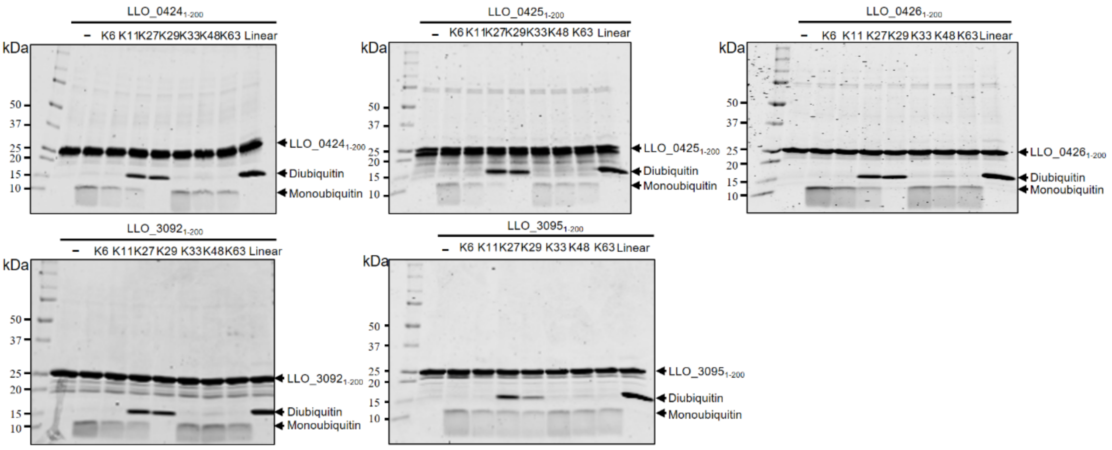

FIG S2. Hydrolysis of diubiquitins by the DUB modules of the *L. longbeachae* SidE family proteins. The DUB domains of SidE orthologue proteins in *L. longbeachae* were purified and incubated with a panel of diubiquitins at 37 °C for 2 h. Cleavage of diubiquitins were visualized after SDS-PAGE followed by Coomassie brilliant blue staining (CBB). Data shown are one representative from three independent experiments.

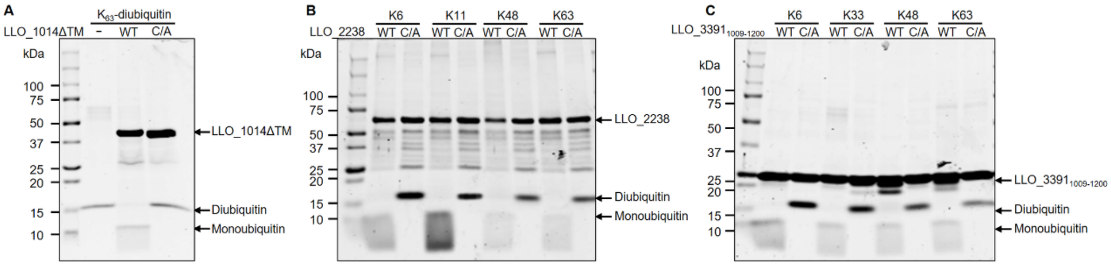

FIG S3. Cleavage of diubiquitins requires the catalytic cysteine in DUBs. The indicated diubiquitins were incubated with WT or their active cysteine mutant DUBs at 37 °C for 1 h. Proteins in the reactions were separated by SDS-PAGE and detected by CBB. Data shown are one representative from three independent experiments.

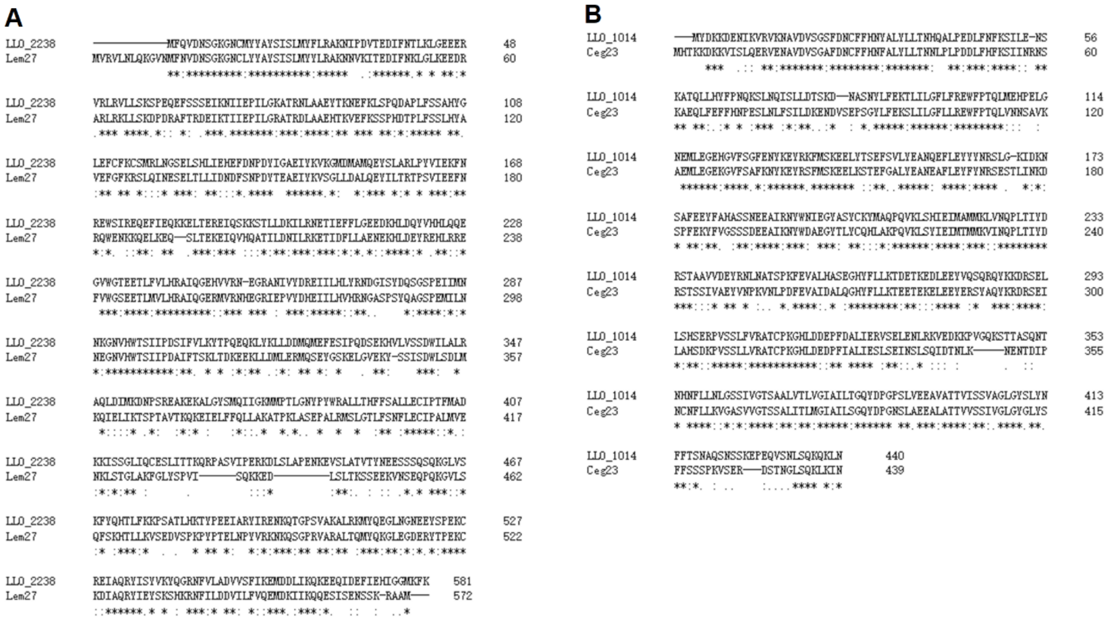

FIG S4. Sequence alignments of *L. longbeachae* OTU proteins and their orthologs in *L. pneumophila*. The alignments are generated via Clustal Omega (https://www.ebi.ac.uk/Tools/msa/clustalo/) using LLO_2238/Lem27 (A) or LLO_1014/Ceg23 (B) as queries.

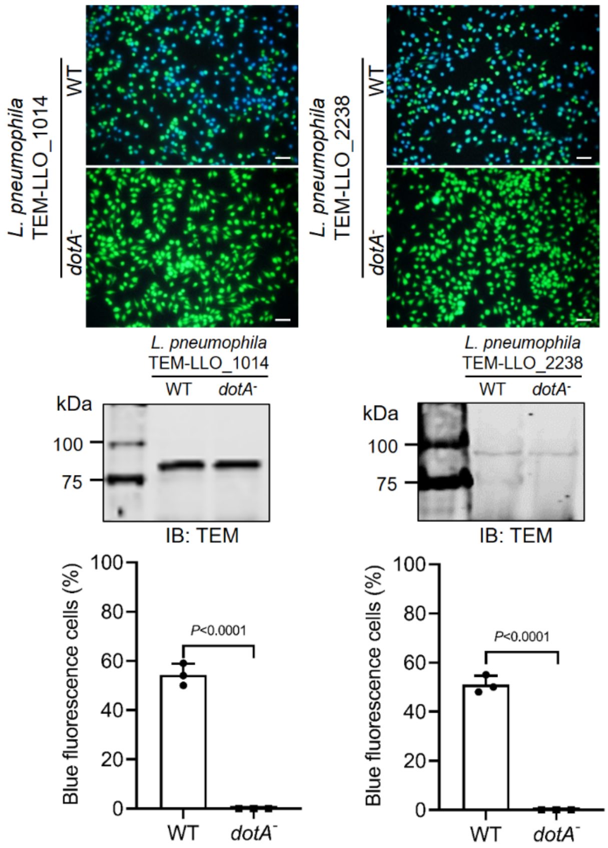

FIG S5. Translocation of *L. longbeachae* OTUs by the *L. pneumophila* Dot/Icm system. Plasmids expressing the indicated TEM fusion proteins were electroporated into both wild-type *L. penumophila* and *dotA*$^{-}$ mutant. These strains were used to infect RAW264.7 cells for 2 h at an MOI of 50. After loading the samples with CCF4/AM, translocation of the TEM-fused OTUs were visualized by the Olympus IX83 fluorescence microscope equipped with a β-lactamase FL-Cube. Scale bar: 100 μm. Expression of the TEM fusion proteins in *L. pneumophila* strains were confirmed by probing the bacterial cells with a TEM antibody. Three randomly selected images were used to count the cells emitting blue fluorescence signals (n = 500). Data are one representative of three independent experiments.

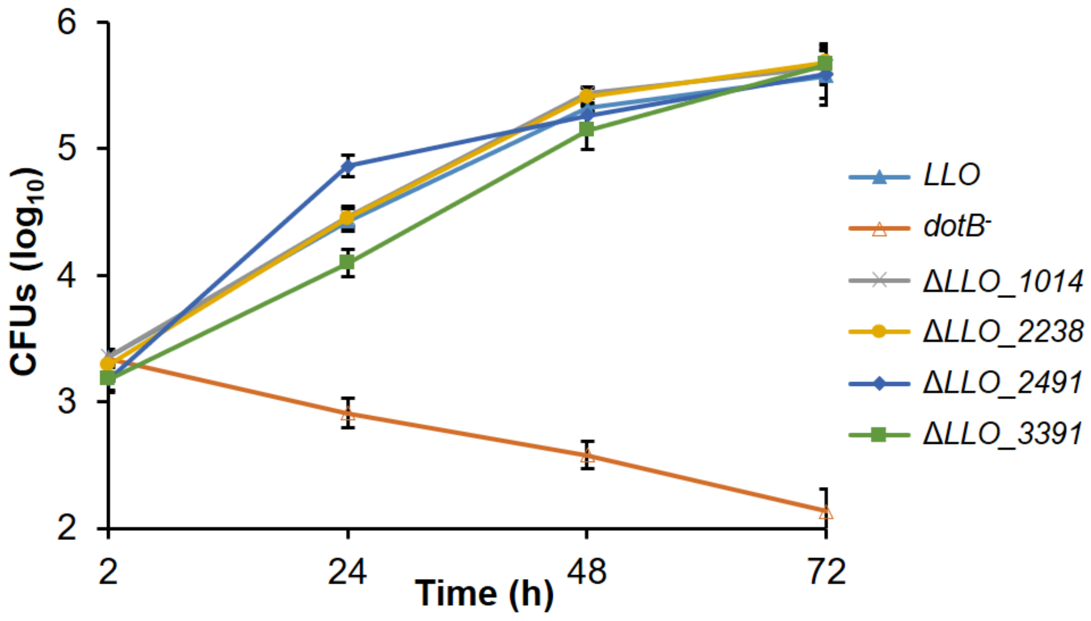

FIG S6. *L. longbeachae* DUBs are not required for bacterial intracellular replication. U937 cells were challenged with wild-type, Δ*dotB*, Δ*LLO_1014*, Δ*LLO_2238*, Δ*LLO_2491*, and Δ*LLO_3391* at an MOI of 10. At indicated time after infection, infected cells were lysed and plated on CYE plates. The CFUs were counted after culturing at 37 °C for 4 days. Results are presented as mean ± SD and are one representative from three independent assays done in triplicate.

Table S1. Bacterial strains used in the study.

Bacterial Strains	Relevant properties	Reference
E. coli		
DH5α(λpir)	supE44 d*lacU169*(φ80*lacZ*Δ*M15) hsdR17 recA1 endA1 gyrA96 thi-1 relA1 pir tet::Mu recA*	Our collection
BL21(DE3)	F^- *omp*T *hsd*SB (rB^- mB^-) *gal dcm* (DE3)	Our collection
L. longbeachae		
L. longbeachae serogroup 1	ATCC[a] 33462; type strain	ATCC 33462
YS0001	ATCC 33462 *L. longbeachae* serogroup 1 strain with *dotB*⁻ deletion mutation	This study
YS0002	LLO+pXDC61JQ-Flag	This study
YS0003	LLO *dotB*⁻+pXDC61JQ-Flag	This study
YS0004	LLO Δ*LLO_1014*	This study
YS0005	LLO Δ*LLO_1014*+pXDC61JQ-Flag	This study
YS0006	LLO Δ*LLO_1014*+pXDC61JQ-Flag-LLO_1014	This study
YS0007	LLO Δ*LLO_1014*+pXDC61JQ-Flag-LLO_1014_{C26A}	This study
YS0008	LLO Δ*LLO_2238*	This study
YS0009	LLO Δ*LLO_2238*+pXDC61JQ-Flag	This study
YS0010	LLO Δ*LLO_2238*+pXDC61JQ-Flag-LLO_2238	This study
YS0011	LLO Δ*LLO_2238*+pXDC61JQ-Flag-LLO_2238_{C12A}	This study
YS0012	LLO Δ*LLO_2491*	This study
YS0013	LLO Δ*LLO_3391*	This study
YS0014	LLO+pXDC61JQ-Flag-LLO_1014	This study
YS0015	LLO *dotB*⁻+pXDC61JQ-Flag-LLO_1014	This study
YS0016	LLO+pXDC61JQ-Flag-LLO_2238	This study
YS0017	LLO *dotB*⁻+pXDC61JQ-Flag-LLO_2238	This study
L. pneumophila		
Lp02	Philadelphia-1 *rpsL hsdR thyA*	(1)
Lp03	Lp02 *dotA*⁻	(1)
Lp02 (pZL507)	Lp02+pZL507	(2)
Lp03 (pZL507)	Lp03+pZL507	(2)
YS0018	LP02 Δ*ceg23*	(3)
YS0019	LP02 Δ*ceg23*+pZL507	(3)
YS0020	LP02 Δ*ceg23*+pZL507-Ceg23	(3)
YS0021	LP02 Δ*ceg23*+pZL507-LLO_1014	This study
YS0022	LP02 Δ*ceg23*+pZL507-LLO_1014_{C26A}	This study
YS0023	LP02 Δ*lem27*	(4)

YS0024	LP02 Δ*lem27*+pZL507	(4)
YS0025	LP02 Δ*lem27*+pZL507-Lem27	(4)
YS0026	LP02 Δ*lem27*+pZL507-LLO_2238	This study
YS0027	LP02 Δ*lem27*+pZL507-LLO_2238$_{C12A}$	This study
YS0028	LP02 TEM-*LLO_1014*	This study
YS0029	Lp03 *dotA*$^{-}$ TEM-*LLO_1014*	This study
YS0030	LP02 TEM-*LLO_2238*	This study
YS0031	Lp03 *dotA*$^{-}$ TEM-*LLO_2238*	This study

[a] ATCC, American Type Culture Collection

Table S2. Plasmids used in the study.

Plasmids	Properties	Reference
pET28a	Kan^R, *E. Coli* expression vectors for His-tagged proteins	Novagen (CAT#69864)
pET28a-*LLO_0424*$_{1\text{-}200}$	LLO_0424$_{1\text{-}200}$ (LLO_0424 residues 1-200) in pET28a	This study
pET28a-*LLO_0425*$_{1\text{-}200}$	LLO_0425$_{1\text{-}200}$ (LLO_0425 residues 1-200) in pET28a	This study
pET28a-*LLO_0426*$_{1\text{-}200}$	LLO_0426$_{1\text{-}200}$ (LLO_0426 residues 1-200) in pET28a	This study
pET28a-*LLO_3092*$_{1\text{-}200}$	LLO_3092$_{1\text{-}200}$ (LLO_3092 residues 1-200) in pET28a	This study
pET28a-*LLO_3095*$_{1\text{-}200}$	LLO_3095$_{1\text{-}200}$ (LLO_3095 residues 1-200) in pET28a	This study
pET28a-*LLO_ 1014ΔTM*	LLO_1014ΔTM (LLO_1014 residues 1-358) in pET28a	This study
pET28a-*LLO_ 1014ΔTM*$_{C26A}$	pET28a-*LLO_1014ΔTM* with mutation C26A	This study
pET28a-*LLO_2238*	Full length *LLO_2238* in pET28a	This study
pET28a-*LLO_2238*$_{C12A}$	pET28a-*LLO_2238* with mutation C12A	This study
pET28a-*LLO_2491ΔC102*	LLO_2491ΔC102 (LLO_2491 residues 1-270) in pET28a	This study
pET28a-*LLO_3391*$_{1009\text{-}1200}$	LLO_3391$_{1009\text{-}1200}$ (LLO_3391 residues 1009-1200) in pET28a	This study
pET28a-*LLO_3391*$_{1009\text{-}1200\ C1124A}$	pET28a-*LLO_3391*$_{1009\text{-}1200}$ with mutation C1124A	This study
pET28a-*LLO_0794*	Full length *LLO_0794* in pET28a	This study
pET28a-*LLO_1369*	Full length *LLO_1369* in pET28a	This study
pET28a-*LLO_1631*	Full length *LLO_1631* in pET28a	This study
pET28a-*LLO_3118*	Full length *LLO_3118* in pET28a	This study
pET28a-*LLO_2210*	Full length *LLO_2210* in pET28a	This study
pET28a-*LLO_2179*	Full length *LLO_2179* in pET28a	This study
pET28a-*LLO_2985*	Full length *LLO_2985* in pET28a	This study
pET28a-*LLO_2066*	Full length *LLO_2066* in pET28a	This study
pSR47s	R6K suicide vector (Kan^R, sacB)	(5)
pSR47s-Δ*LLO_1014*	pSR47s containing the flanking region of *LLO_1014*	This study
pSR47s-Δ*LLO_2238*	pSR47s containing the flanking region of *LLO_2238*	This study
pSR47s-Δ*LLO_2491*	pSR47s containing the flanking region of *LLO_2491*	This study
pSR47s-Δ*LLO_3391*	pSR47s containing the flanking region of *LLO_3391*	This study

pSR47s-Δ*LLO_2066*	pSR47s containing the flanking region of *LLO_2066*	This study
peGFPC1	For expressing N-terminal GFP fusion proteins in mammalian cells	Clontech
peGFP-$sdeA_{Dub}$	$sdeA_{Dub}$ (residues 1-200) in peGFPC1	(6)
peGFP-*LLO_1014*	*LLO_1014* in peGFPC1	This study
peGFP-LLO_1014_{C26A}	LLO_1014_{C26A} in peGFPC1	This study
peGFP-*LLO_2238*	*LLO_2238* in peGFPC1	This study
peGFP-*LLO_2491*	*LLO_2491* in peGFPC1	This study
peGFP-$LLO\_3391_{1009-1200}$	$LLO\_3391_{1009-1200}$ in peGFPC1	This study
peGFP-*LLO_2066*	*LLO_2066* in peGFPC1	This study
pZL507	For expression His_6-tagged protein *L. pneumophila*	(7)
pZL507-4×Flag-*LLO_1014*	4×Flag- *LLO_1014* in pZL507	This study
pZL507-4×Flag-LLO_1014_{C26A}	4×Flag- LLO_1014_{C26A} in pZL507	This study
pZL507-4×Flag-*LLO_2238*	4×Flag- *LLO_2238* in pZL507	This study
pZL507-4×Flag-LLO_2238_{C12A}	4×Flag-LLO_2238_{C12A} in pZL507	This study
p3×HACDNA3.1-*ub*	Ubiquitin in p3×HACDNA3.1	(6)
p3xHACDNA3.1-*ub-63K*	Ub63K in p3×HACDNA3.1	(4)
pXDC61m	Encodes IPTG-inducible with N-terminal BlaM fusion; Cm^R	(8)
pXDC61m-*LLO_1014*	*LLO_1014* in pXDC61m	This study
pXDC61m-*LLO_2238*	*LLO_2238* in pXDC61m	This study
pXDC61JQ	Encodes IPTG-inducible expressing N-terminal Flag fusion proteins in *L. Longbeachae*; Cm^R	This study
pXDC61JQ-*LLO_1014*	*LLO_1014* in pXDC61JQ	This study
pXDC61JQ-*LLO_2238*	*LLO_2238* in pXDC61JQ	This study

Table S3. Primers used in the study.

Primers	Sequence[a]	Note
pYS1001	CTGGGATCCATGTACGATAAAAAAGATG	*LLO_1014* 5F BamHI
pYS1002	CTGGTCGACTTAATTCAACTTTTGTTT	*LLO_1014* 3R SalI
pYS1003	CTGGTCGACTCAAAGGAAGTTATGATTTG	*LLO_1014ΔTM* 3R SalI
pYS1004	CTGGGATCCATGTTTCAAGTGGATAATAG	*LLO_2238* 5F BamHI
pYS1005	CTGGTCGACTTACTTGAACTTCATTCC	*LLO_2238* 3R SalI
pYS1006	CTGGGATCCATGCCTTTTAGAATAGAT	*LLO_2491* 5F BamHI
pYS1007	CTGGTCGACTTAGTAGCTTAAAATTCG	*LLO_2491* 3R SalI
pYS1008	CTGGTCGACTCAAGAAATTTCCCGGGGT	*LLO_2491ΔC102* 3R SalI
pYS1009	CTGGGATCCATGAAAAGCATTACAGAG	*LLO_2066* 5F BamHI
pYS1010	CTGGTCGACTTACCCGAGTGTTGAAGA	*LLO_2066* 3R SalI
pYS1011	CTGGGATCCATGCCTGAATACATAAAAG	*LLO_0424*$_{1\text{-}200}$ 5F BamHI
pYS1012	CTGGTCGACTTATATTTCTTTTAGAACAGT	*LLO_0424*$_{1\text{-}200}$ 3R SalI
pYS1013	CTGAGATCTATGCCTAAGTATGTAAAAG	*LLO_0425*$_{1\text{-}200}$ 5F BglII
pYS1014	CTGGTCGACTTACGGAAGTACTCTGAGATG	*LLO_0425*$_{1\text{-}200}$ 3R SalI
pYS1015	CTGGGATCCATGCCTAAATACGTAAAAG	*LLO_0426*$_{1\text{-}200}$ 5F BamHI
pYS1016	CTGGTCGACTTAAGGGATTTCTATATGTAA	*LLO_0426*$_{1\text{-}200}$ 3R SalI
pYS1017	CTGGGATCCATGCCAAAATACATAAAAG	*LLO_3092*$_{1\text{-}200}$ 5F BamHI
pYS1018	CTGGTCGACTTAAGTTGGAGTTTCTATATG	*LLO_3092*$_{1\text{-}200}$ 3R SalI
pYS1019	CTGAGATCTATGCCCGAGTTTGTGCAAG	*LLO_3095*$_{1\text{-}200}$ 5F BglII
pYS1020	CTGGTCGACTTATGCGGGTATGTGTATGTT	*LLO_3095*$_{1\text{-}200}$ 3R SalI
pYS1021	CTGGGATCCATGTTATACGTAGAAGAAATG	*LLO_3391*$_{1009\text{-}1200}$ 5F BamHI
pYS1022	CTGGTCGACTCATTCACTTTCTTGCCTTA	*LLO_3391*$_{1009\text{-}1200}$ 3R SalI
pYS1023	CTGGGATCCATGATTCCAGTAGAAATAG	*LLO*_1631 5F BamHI
pYS1024	CTGGTCGACTTAAGAAACGGAGCCCAT	*LLO*_1631 3R SalI
pYS1025	CTGGGATCCATGCGACTAGAGATATCAA	*LLO*_3118 5F BamHI
pYS1026	CTGGTCGACTTATCGATTATGCTTTAT	*LLO*_3118 3R SalI
pYS1027	CTGGAGCTCATGAAAGATGATAAGTCAG	*LLO*_2179 5F SacI
pYS1028	CTGGCGGCCGCTTAAACTTTAAATTGGCT	*LLO*_2179 3R NotI
pYS1029	CTGGGATCCATGCAAAACTCATGGAAAG	*LLO*_0794 5F BamHI
pYS1030	CTGGTCGACTCATTTCTCTGCACTAAT	*LLO*_0794 3R SalI
pYS1031	CTGGCGGCCGCATGAGAGATGATAAGTTAG	*LLO*_2985 5F NotI
pYS1032	CTGCTCGAGCTAAAAGTTAAATTGTCT	*LLO*_2985 3R XhoI
pYS1033	CTGGGATCCATGTTTACCGAAGAATTCA	*LLO*_1369 5F BamHI
pYS1034	CTGGTCGACTTAGACACTTGTAACGTT	*LLO*_1369 3R SalI
pYS1035	CTGGCGGCCGCATGACTCTACCAATATTT	*LLO*_2210 5F NotI
pYS1036	CTGGTCGACTCATTTCAAATGAATCCC	*LLO*_2210 3R SalI
pYS1107	CTGGTCGACTACCAAGCTGGTAAGA	*LLO dotB*$^-$ knockout up SalI-F
pYS1108	ATTAATTTATATACTCGAGTAAAACGAGTAGGCTC ATCAGG	*LLO dotB*$^-$ knockout up-R
pYS1109	CCTGATGAGCCTACTCGTTTTACTCGAGTATATAA ATTAAT	*LLO dotB*$^-$ knockout down-F
pYS1110	CTGGGATCCGGTAAGGGGGATGAAT	*LLO dotB*$^-$ knockout down

		BamHI-R
pYS1037	ACGCGTCGACAACCCGCTCATTATGTTACC	*LLO_1014* knockout up SalI-F
pYS1038	AAATTGGATACTTGTTCTGGATTTTTACTCGAACTTTAA	*LLO_1014* knockout up-R
pYS1039	TTAAAGTTCGAGTAAAAAATCCAGAACAAGTATCCAATTT	*LLO_1014* knockout down-F
pYS1040	CGCGGATCCTAATCGATTTTTTTAGCAGG	*LLO_1014* knockout down BamHI-R
pYS1041	ACGCGTCGACACTCACAATGTTTTCCTATT	*LLO_2238* knockout up SalI-F
pYS1042	TGCTCAATAAACTCATCAATATAATACATACAATTGCCTT	*LLO_2238* knockout up-R
pYS1043	AAGGCAATTGTATGTATTATATTGATGAGTTTATTGAGCA	*LLO_2238* knockout down-F
pYS1044	CGCGGATCCAAATTTATCATTTGTAGGAA	*LLO_2238* knockout down BamHI-R
pYS1045	ACGCGTCGACTTTAGAAGATGATACTGGTG	*LLO_2491* knockout up SalI-F
pYS1046	TCTGCTTCTTGTAAGCGGGTAAATCCACAATCACCACCAC	*LLO_2491* knockout up-R
pYS1047	GTGGTGGTGATTGTGGATTTACCCGCTTACAAGAAGCAGA	*LLO_2491* knockout down-F
pYS1048	CGCGGATCCAAAAGTTCCTTAAGTCAATG	*LLO_2491* knockout down BamHI-R
pYS1049	ACGCGTCGACGCTTTAATAAATCCCTCAAG	*LLO_3391* knockout up SalI-F
pYS1050	TTCTGTTCAGCATTAGTGTCTTTTTCCTTAGAACCTTCGT	*LLO_3391* knockout up-R
pYS1051	ACGAAGGTTCTAAGGAAAAAGACACTAATGCTGAACAGAA	*LLO_3391* knockout down-F
pYS1052	CGCGGATCCTCTGTCTCTGTTTTCATCCA	*LLO_3391* knockout down BamHI-R
pYS1053	AAGAAATACTATACGCATAATACATAGCATTGCCTTTACCACTATTATCCACTTG	*LLO_2238*$_{C12A}$-1
pYS1054	CAAGTGGATAATAGTGGTAAAGGCAATGCTATGTATTATGCGTATAGTATTTCTT	*LLO_2238*$_{C12A}$-2
pYS1055	AGTAAATAAAGTGCATAGTTATGAAAAAAAGCATTATCAAAACTTCCAGAAACATCAACTGC	*LLO_1014*$_{C26A}$-1
pYS1056	GCAGTTGATGTTTCTGGAAGTTTTGATAATGCTTTTTTTCATAACTATGCACTTTATTTACT	*LLO_1014*$_{C26A}$-2
pYS1057	GCAACCCAATACCCAGCTAATCCTCTATTTAATCCTTTGGCATTCG	*LLO_3391*$_{C1124A}$-1
pYS1058	CGAATGCCAAAGGATTAAATAGAGGATTAGCTGGGTATTGGGTTGC	*LLO_3391*$_{C1124A}$-2

[a] Restriction enzyme sites are underlined.

REFERENCES

1. Berger KH, Isberg RR. 1993. Two distinct defects in intracellular growth complemented by a single genetic locus in *Legionella pneumophila*. *Mol Microbiol* 7:7-19 https://doi.org/10.1111/j.1365-2958.1993.tb01092.x.

2. Liu Y, Luo ZQ. 2007. The *Legionella pneumophila* effector SidJ is required for efficient recruitment of endoplasmic reticulum proteins to the bacterial phagosome. *Infect Immun* 75:592-603 https://doi.org/10.1128/iai.01278-06.

3. Ma K, Zhen X, Zhou B, Gan N, Cao Y, Fan C, Ouyang S, Luo ZQ, Qiu J. 2020. The bacterial deubiquitinase Ceg23 regulates the association of Lys-63-linked polyubiquitin molecules on the *Legionella* phagosome. *J Biol Chem* 295:1646-1657 https://doi.org/10.1074/jbc.RA119.011758.

4. Liu S, Luo J, Zhen X, Qiu J, Ouyang S, Luo ZQ. 2020. Interplay between bacterial deubiquitinase and ubiquitin E3 ligase regulates ubiquitin dynamics on *Legionella* phagosomes. *Elife* 9:e58114 https://doi.org/10.7554/eLife.58114.

5. Duménil G, Isberg RR. 2001. The *Legionella pneumophila* IcmR protein exhibits chaperone activity for IcmQ by preventing its participation in high-molecular-weight complexes. *Mol Microbiol* 40:1113-27 https://doi.org/10.1046/j.1365-2958.2001.02454.x.

6. Qiu J, Sheedlo MJ, Yu K, Tan Y, Nakayasu ES, Das C, Liu X, Luo ZQ. 2016. Ubiquitination independent of E1 and E2 enzymes by bacterial effectors. *Nature* 533:120-4 https://doi.org/10.1038/nature17657.

7. Xu L, Shen X, Bryan A, Banga S, Swanson MS, Luo ZQ. 2010. Inhibition of host vacuolar H+-ATPase activity by a *Legionella pneumophila* effector. *PLoS*

Pathog 6:e1000822 https://doi.org/10.1371/journal.ppat.1000822.

8. Zhu W, Banga S, Tan Y, Zheng C, Stephenson R, Gately J, Luo ZQ. 2011. Comprehensive identification of protein substrates of the Dot/Icm type IV transporter of *Legionella pneumophila*. *PLoS One* 6:e17638 https://doi.org/10.1371/journal.pone.0017638.
